# Integrated Time- and Frequency-Domain Reflectometry for Non-Invasive Moisture Monitoring in Historic Masonry Materials

**DOI:** 10.3390/s26185856

**Published:** 2026-09-16

**Authors:** Giuseppe Cannazza, Andrea Cataldo, Raissa Schiavoni, Giovanni Leucci, Antonio Masciullo, Dora Francesca Barbolla, Lara De Giorgi

**Affiliations:** 1 Institute of Heritage Science (National Research Council), 73100 Lecce, Italy; giuseppe.cannazza@cnr.it (G.C.); giovanni.leucci@cnr.it (G.L.); dorafrancesca.barbolla@cnr.it (D.F.B.); lara.degiorgi@cnr.it (L.D.G.); 2 Department of Engineering for Innovation, University of Salento, 73100 Lecce, Italy; antonio.masciullo@unisalento.it

**Keywords:** time-domain reflectometry, frequency-domain reflectometry, moisture monitoring, historic masonry, patch antenna, microstrip probe, non-destructive testing, cultural heritage

## Abstract

Moisture monitoring in historic masonry requires non-destructive solutions that can be adapted to the characteristics and value of the asset, the spatial extent of the phenomenon, and the need for repeated measurements over time. This work presents an integrated reflectometric methodology that combines accurate Frequency-Domain Reflectometry (FDR) characterization with the development and validation of a custom-developed, compact, portable, low-cost, and potentially scalable Time-Domain Reflectometry (TDR) prototype. Resonant patch antennas operating at different frequencies were used with a Vector Network Analyzer (VNA) to characterize Lecce stone, Carparo, and plaster applied to Carparo during controlled drying. This configuration provides localized moisture information based on sensor–material-specific calibration and is particularly suitable for valuable architectural, monumental, or decorative surfaces, where measurement accuracy and detailed spectral analysis are essential. The proposed TDR prototype extends the same reflectometric approach toward more accessible, low-cost, repeated, distributed, and potentially remotely managed monitoring. Its performance was assessed against a reference PicoVNA using a microstrip probe, selected as a representative application for monitoring broader masonry regions and phenomena such as rising damp. The reflectograms acquired with the prototype were processed to reconstruct the corresponding complex $S_{11}\left(f\right)$ responses, allowing the two measurement systems to be compared on a common frequency-domain basis. The results showed a consistent moisture-dependent resonance evolution and demonstrated that the prototype preserves the principal temporal and spectral information observed with the reference instrument. The proposed methodology therefore brings together complementary measurement capabilities within a unified framework, enabling the sensing configuration to be selected according to the required accuracy, spatial coverage, portability, cost, scalability, and monitoring frequency.

## 1. Introduction

Moisture is one of the most persistent causes of deterioration in historic masonry [1,2]. Water entering through capillary rise, wind-driven rain [3,4,5,6], infiltration, condensation, or defective drainage [7] can remain trapped within the porous network of stone, mortar, and plaster, promoting salt transport [8] and crystallization [9,10,11], biological colonization [12], freeze–thaw damage, mechanical disintegration [13], and the detachment of plaster [14] and decorative layers [15,16]. Reliable moisture assessment [17] is therefore essential for identifying active degradation processes, planning conservation interventions [18], evaluating their effectiveness, and supporting preventive maintenance [19] through repeated measurements over time [20]. In cultural-heritage structures, however, such monitoring must be performed without removing or permanently altering the original material.

Conventional reference methods, such as gravimetric analysis [21] and core sampling, provide quantitative information but are destructive [22] and cannot be repeatedly applied at the same location. Several non-destructive techniques have consequently been investigated, including infrared thermography [23], electrical-resistivity measurements [24], nuclear magnetic resonance [25], ground-penetrating radar [26,27], and dielectric and microwave methods [28,29,30]. Recent studies have also investigated complementary non-destructive and minimally invasive approaches for masonry and stone moisture assessment, including comparative multi-method monitoring, electrical-tomography-based reconstruction, and optical-fiber sensing [31,32,33]. Their practical suitability depends not only on sensitivity and investigation depth, but also on material heterogeneity, environmental conditions, calibration requirements, instrumentation cost, repeatability, portability, and operator expertise. These aspects become particularly important when the objective is not a single diagnostic inspection, but repeated, spatially distributed, or remotely managed monitoring.

In this context, electromagnetic reflectometric methods [34] are especially attractive because water strongly modifies the effective dielectric properties of porous mineral materials [28,35]. Moisture variations therefore affect propagation velocity, attenuation, characteristic impedance, and reflection behavior [36,37,38]. These effects can be observed either in the frequency domain, through the complex reflection coefficient $S_{11}\left(f\right)$, or in the time domain, through the reflectogram generated by a fast-rising excitation. Although the two representations arise from the same interaction between the sensing structure and the investigated material, they are generally acquired using different instruments and interpreted through different parameters.

Frequency-Domain Reflectometry (FDR) measurements performed using a Vector Network Analyzer (VNA) provide accurate and stable spectral information and are particularly effective when resonant sensing elements are employed. An increase in moisture generally increases the effective permittivity seen by the sensor, producing a measurable shift in resonance frequency together with changes in resonance depth and bandwidth [28]. By modifying the geometry of a patch antenna, its operating frequency can be selected and the sensing element adapted to different material configurations and diagnostic requirements. Resonant sensors combined with a VNA are therefore well suited to accurate, localized, and material-specific moisture characterization [39,40,41]. Such an approach is particularly valuable for architecturally significant surfaces, museum environments, decorative elements, and other applications in which measurement accuracy and controlled localization are primary requirements.

However, even portable VNAs remain comparatively expensive and may be impractical for permanent installation, dense measurement networks, or continuous monitoring of extended masonry surfaces. In these scenarios, a more compact and cost-effective acquisition system is desirable, particularly for following widespread phenomena such as rising damp. Time-Domain Reflectometry (TDR) represents a promising alternative because it relies on relatively simple pulse-generation and acquisition electronics [36,37]. In TDR, a fast-rising voltage step propagates along a transmission structure coupled to the material, and moisture-induced variations in dielectric permittivity modify the delay, amplitude, and shape of the reflected waveform [38]. Originally developed for soil-moisture assessment [37,42,43], TDR has subsequently been applied to porous construction materials [44,45] and masonry using non-invasive and flat probe geometries [46,47], as well as to diffused and spatially extended reflectometric monitoring architectures [48].

Several methodological elements underlying the present study were established in previous works and should therefore be distinguished from the specific contribution of this work. Earlier studies [49,50] developed time-to-frequency-domain processing procedures for reconstructing frequency-dependent reflectometric information from TDR measurements, primarily for dielectric characterization and quality monitoring of liquids. In the field of Cultural Heritage materials, Ref. [39] compared different microwave reflectometric probes for water-content estimation in stone materials and included the reconstruction of the frequency-domain response of a patch resonator from measurements acquired using a commercial TDR. In addition, Ref. [48] investigated elongated TDR sensing elements for diffused monitoring applications, including building structures. Thus, the individual principles of TDR, resonant FDR, and TD–FD transformation are not claimed as novel contributions of the present work. Table 1 summarizes the main differences between these previous studies and the present investigation.

Against this background, the contribution of the present work lies in the integration and experimental validation of these established reflectometric principles within a unified framework specifically developed for moisture monitoring in historic masonry, rather than in the introduction of the individual TDR, FDR, or TD–FD techniques themselves. The first distinctive element is a multi-frequency FDR characterization performed using two contact-based resonant antennas on three masonry configurations, including a layered plaster-on-Carparo system, resulting in six sensor–material datasets and corresponding moisture–calibration relationships. The second is the development and experimental implementation of a dedicated compact and low-cost portable TDR prototype, combined with an elongated planar microstrip probe intended for broader masonry regions and rising-damp applications. Finally, the two branches are connected through a common validation procedure in which the prototype response is compared with the PicoVNA measurements in both the time and frequency domains and, ultimately, through the corresponding moisture–resonance calibration relationships. The advancement of the present work therefore resides in this masonry-oriented system integration and cross-domain validation, together with the dedicated portable instrumentation and sensing configurations.

The experimental activity is organized into two connected branches. In the first branch, two contact-based patch antennas, denoted as P1 and P2 and designed to operate at different resonance frequencies, are used with a PicoVNA 108 to characterize Lecce stone, Carparo, and plaster on Carparo during controlled drying. The resulting six material–antenna datasets are used to establish relationships between resonance frequency and gravimetric moisture, thereby demonstrating the sensitivity and flexibility of resonant frequency-domain sensing across different masonry configurations.

The second branch focuses on the developed TDR prototype, which integrates a fast step generator, portable acquisition electronics, and dedicated software for waveform acquisition, storage, calibration, and time-to-frequency-domain reconstruction. Its validation is performed using a dedicated microstrip probe, denoted as M1. The elongated and scalable geometry of M1 is particularly relevant to rising-damp applications, since the sensing structure can be extended longitudinally along a wall section and used to investigate a broader vertical region than a localized patch antenna. The experiments are conducted on Carparo, a porous calcarenitic stone widely used in historic and traditional masonry in Southern Italy and representative of structures affected by rising damp. The prototype is evaluated against the PicoVNA 108 through complementary time- and frequency-domain comparisons, including moisture-dependent reflectograms, reconstructed $S_{11}\left(f\right)$ responses, and resonance-based calibration relationships.

Rather than claiming full metrological equivalence with the reference instrument, the study assesses whether the developed prototype can reproduce the principal temporal and spectral features associated with moisture variation, including resonance location, moisture-dependent resonance shift, and calibration trend. In this way, the proposed framework combines the accuracy of VNA-based spectral characterization with the portability, reduced cost, and scalability of the developed TDR system.

The remainder of the paper is organized as follows. Section 2 introduces the relevant reflectometric principles and describes the TDR-to-frequency-domain reconstruction procedure. Section 3 presents the masonry specimens, sensing structures, instrumentation, moisture-conditioning protocol, experimental workflow, and data-analysis methods. Section 4 reports the FDR moisture-dependent responses and calibration curves, followed by the time- and frequency-domain validation of the portable TDR prototype. Finally, Section 5 summarizes the principal findings, discusses their practical implications, and outlines future developments.

## 2. Measurement Principles and Processing Framework

The proposed framework exploits the sensitivity of electromagnetic reflections to moisture-induced variations in the dielectric properties of porous materials. As water progressively replaces air within the pore network, the effective complex permittivity of the material changes, thereby modifying wave-propagation velocity, attenuation, characteristic impedance, and reflection behavior. These effects can be observed either in the frequency domain, through variations in the complex reflection coefficient $S_{11}\left(f\right)$, or in the time domain, through changes in the amplitude, shape, and propagation delay of the reflectogram.

Although the time- and frequency-domain representations originate from the same sensor–material interaction, they provide different observables. Frequency-domain measurements allow moisture-induced variations in resonance frequency and spectral shape to be evaluated directly, whereas time-domain measurements describe changes in reflection amplitude, waveform shape, and propagation delay. In the present study, the prototype reflectograms are also processed and calibrated to reconstruct the corresponding complex $S_{11}\left(f\right)$ response. The reconstructed spectra can therefore be compared directly with those measured using the PicoVNA in terms of resonance position, spectral evolution, and moisture-calibration behavior.

### 2.1. Frequency-Domain Reflectometry

FDR characterizes the interaction between a sensing structure and the investigated material by measuring the complex reflection coefficient $S_{11}\left(f\right)$ over a selected frequency range. When a resonant element, such as a patch antenna or a microstrip resonator, is placed in contact with a porous material, part of its electromagnetic field extends into the sample. Consequently, both the resonance frequency and the overall spectral response depend on the effective dielectric properties of the combined sensor–material system.

For a simplified resonant structure, the resonance frequency can be approximated as

(1) $$f_{\mathrm{res}}\approx \frac{c}{2L_{\mathrm{eff}}\sqrt{\varepsilon_{\mathrm{eff}}}},$$

where *c* is the speed of light, $L_{\mathrm{eff}}$ is the effective resonant length, and $\varepsilon_{\mathrm{eff}}$ is the effective permittivity experienced by the electromagnetic field. As moisture increases, water progressively replaces air within the pore network and the effective permittivity generally rises. This variation typically shifts the resonance toward lower frequencies and may also modify the resonance depth, bandwidth, and phase response because dielectric losses and electrical conductivity change together with permittivity.

The complex coefficient $S_{11}\left(f\right)$ describes the fraction of the incident electromagnetic signal reflected by the sensing structure. Its magnitude and phase therefore contain information on the electromagnetic interaction between the sensor and the investigated material. Moisture-induced variations in the sample may affect not only the resonance frequency $f_{\mathrm{res}}$, but also the overall shape of the spectral response.

Because the resonance shift is often regular over a controlled moisture range, calibration relationships can be derived between $f_{\mathrm{res}}$ and gravimetric moisture. These relationships provide a practical basis for non-destructive moisture assessment, although they remain specific to the sensor geometry, operating frequency, surface condition, contact quality, and material composition. In the present study, the resonance frequency extracted from $|S_{11}\left(f\right)|$ is used as the primary FDR descriptor.

### 2.2. Time-Domain Reflectometry

TDR is a dielectric sensing technique based on the propagation of a fast-rising electromagnetic signal along a transmission line or sensing probe coupled to the material under investigation [36,37,38]. Variations in characteristic impedance, caused by changes in geometry or dielectric properties along the propagation path, generate partial reflections of the incident signal. The analysis of these reflections provides information on the location and magnitude of the corresponding electromagnetic discontinuities and can consequently be used to estimate moisture-dependent changes in the investigated material [38,42,43].

The output of a TDR measurement is the reflectogram, which represents the reflected signal as a function of propagation time. It can be expressed in terms of the normalized time-domain reflection coefficient

(2) $$\rho \left(t\right)=\frac{v_{\mathrm{refl}}\left(t\right)}{v_{\mathrm{inc}}\left(t\right)},$$

where $v_{\mathrm{inc}}\left(t\right)$ and $v_{\mathrm{refl}}\left(t\right)$ are the incident and reflected voltages, respectively. The reflectogram may contain an initial reflection associated with the connection between the instrument and the sensing structure, followed by additional features generated by impedance variations along the probe and by the interaction with the material under test.

For interpretation purposes, the propagation time is often converted into an apparent distance $d_{\mathrm{app}}$, corresponding to the distance that the electromagnetic signal would travel in vacuum during the same time interval. The actual physical distance $d_{\mathrm{real}}$ along the sensing structure is related to the apparent distance through the apparent permittivity $\varepsilon_{\mathrm{app}}$ according to

(3) $$d_{\mathrm{real}}=\frac{d_{\mathrm{app}}}{\sqrt{\varepsilon_{\mathrm{app}}}},$$

and, when the reflection associated with the end of a probe of known physical length $d_{\mathrm{probe}}$ can be identified, the apparent permittivity may be estimated as [36,43]

(4) $$\varepsilon_{\mathrm{app}}={\left(\frac{d_{\mathrm{app}}}{d_{\mathrm{probe}}}\right)}^{2}.$$

As moisture increases, water progressively replaces air within the pore network, generally increasing the effective permittivity of the sensor–material system. The resulting reduction in propagation velocity modifies the temporal position of the reflected features, while changes in impedance and dielectric losses may also affect the amplitude and shape of the reflectogram. These moisture-dependent variations can be interpreted directly in the time domain or used, after suitable processing and calibration, to reconstruct the corresponding frequency-domain response.

### 2.3. Reconstruction of $S_{11}\left(f\right)$ from TDR Measurements

**Figure 1:**
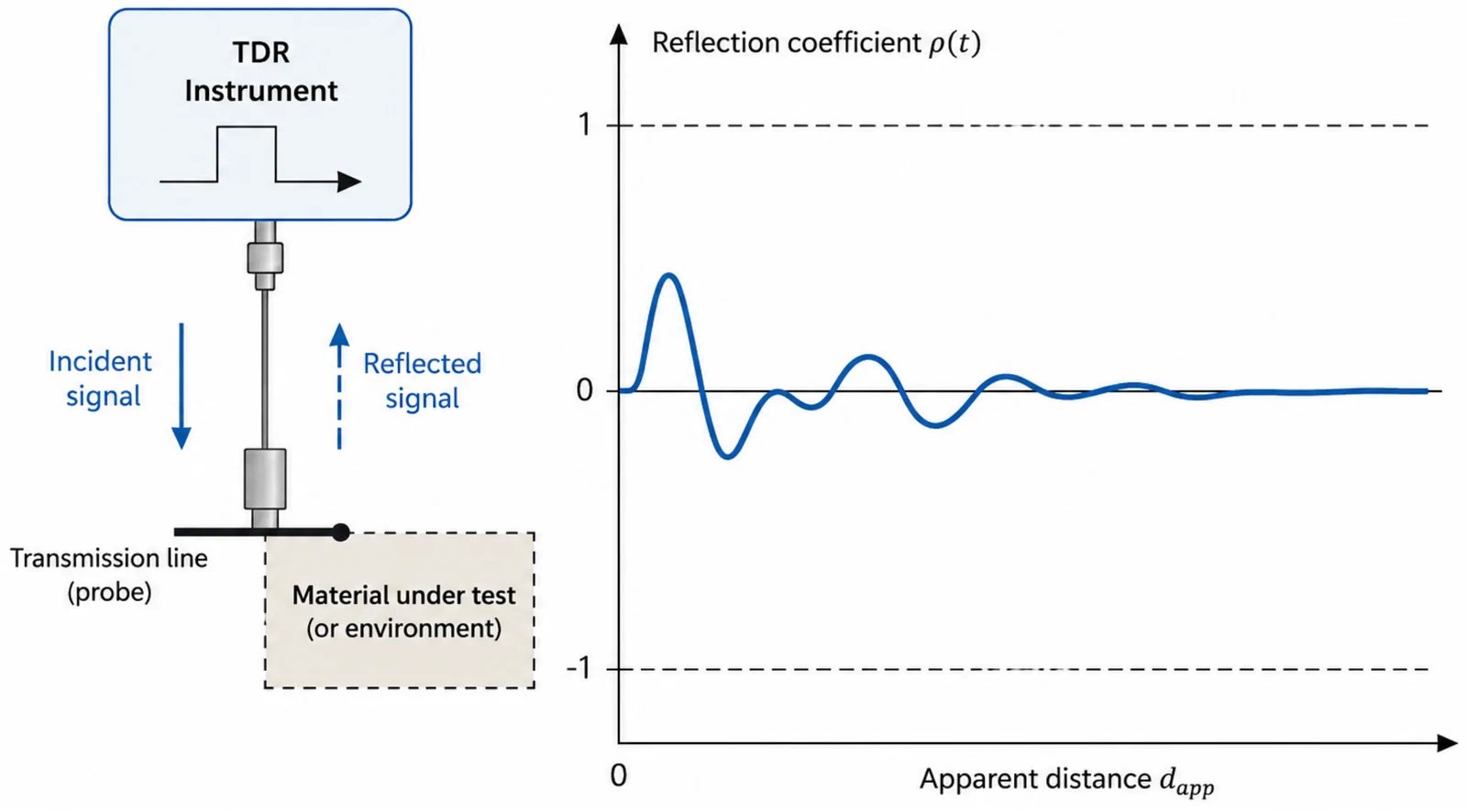
Schematic representation of the TDR measurement principle. A TDR instrument launches an incident signal along the sensing transmission line (probe), and the reflected signal generated by impedance discontinuities associated with the material under test is acquired and represented as the reflection coefficient ρ(t). The corresponding TDR response can also be expressed as a function of the apparent distance $d_{\mathrm{app}}$.

Although TDR measurements are acquired in the time domain, the measured reflectogram contains broadband information that can be transformed into the frequency domain. In the present study, this transformation establishes the link between the portable TDR prototype and the reference VNA, since it allows the response reconstructed from the time-domain waveform to be expressed in terms of the same complex reflection coefficient $S_{11}\left(f\right)$ measured directly by the VNA. The aim is therefore not merely to represent the TDR signal in a different domain, but to verify whether the portable system preserves the moisture-dependent spectral information conventionally observed through frequency-domain instrumentation [49,50]. The TDR measurement principle is schematically illustrated in Figure 1.

Let r[n] denote the sampled and preprocessed reflectogram acquired with sampling interval Δt over a total observation window T=NΔt, where *N* is the number of samples. The natural frequency resolution associated with the observation window is

(5) $$\Delta f=\frac{1}{T}.$$

When a finer frequency grid is required for visualization or feature extraction, the sequence may be extended by zero-padding before Fourier transformation. This operation increases the number of evaluated frequency points and provides a smoother representation of the spectrum, although it does not add information to the original waveform or improve the intrinsic spectral resolution.

Before transformation, the measured reflectogram is normalized to remove the baseline offset introduced by the non-ideal step generator and to ensure that the step-like excitation starts from a common reference level. The Nicolson ramp correction is then applied to reduce spectral leakage and truncation errors associated with the finite observation window before performing the discrete Fourier transform (DFT) [51,52]. The corrected discrete-time sequence is subsequently transformed into the frequency domain according to

(6) R[k]=DFT{r[n]},

where R[k] is the resulting complex discrete spectrum. Only the positive-frequency portion within the usable bandwidth of the measurement system is retained for the subsequent processing.

To reconstruct a calibrated reflection coefficient, the same normalization, Nicolson correction, and Fourier-transformation procedure is applied to the waveforms acquired for the open, short, load, and device-under-test (DUT) conditions. The corresponding complex spectra are denoted as

(7) $$R_{\mathrm{open}}\left[k\right],\;R_{\mathrm{short}}\left[k\right],\;R_{\mathrm{load}}\left[k\right],\;R_{\mathrm{DUT}}\left[k\right].$$

The one-port Short–Open–Load (SOL) calibration implemented in the prototype software is based on a three-term error-correction model. The complex frequency-domain responses obtained from the Open, Short, and Load standards are used to calculate three frequency-dependent error coefficients, following the calibration formulation previously adopted for the reconstruction of frequency-domain responses from TDR measurements [49,50]. The coefficients are given by

(8) $$A_{\mathrm{err}}\left[k\right]=R_{\mathrm{load}}\left[k\right],$$

(9) $$B_{\mathrm{err}}\left[k\right]=\frac{R_{\mathrm{open}}\left[k\right]+R_{\mathrm{short}}\left[k\right]-2R_{\mathrm{load}}\left[k\right]}{R_{\mathrm{open}}\left[k\right]-R_{\mathrm{short}}\left[k\right]},$$

(10) $$C_{\mathrm{err}}\left[k\right]=\frac{2\left(R_{\mathrm{load}}\left[k\right]-R_{\mathrm{open}}\left[k\right]\right)\left(R_{\mathrm{short}}\left[k\right]-R_{\mathrm{load}}\left[k\right]\right)}{R_{\mathrm{open}}\left[k\right]-R_{\mathrm{short}}\left[k\right]}.$$

The calibrated complex reflection coefficient of the DUT is subsequently obtained by applying these error-correction coefficients to the measured DUT spectrum as

(11) $$S_{11}^{\mathrm{DUT}}\left[k\right]=\frac{R_{\mathrm{DUT}}\left[k\right]-A_{\mathrm{err}}\left[k\right]}{B_{\mathrm{err}}\left[k\right]R_{\mathrm{DUT}}\left[k\right]-A_{\mathrm{err}}\left[k\right]B_{\mathrm{err}}\left[k\right]+C_{\mathrm{err}}\left[k\right]}.$$

The final result is the calibrated complex reflection coefficient $S_{11}\left(f_{k}\right)$, from which the corresponding magnitude and phase are obtained as

(12) $$\left|S_{11}\left(f_{k}\right)\right|\;\mathrm{and}\;∠S_{11}\left(f_{k}\right).$$

The reconstructed frequency-domain response can then be compared directly with the $S_{11}\left(f\right)$ measured by the VNA over the common usable frequency range. This comparison makes it possible to determine whether the TDR-based processing chain reproduces the principal moisture-dependent resonance behavior observed with the reference instrument and, consequently, whether the portable prototype retains the spectral information required for moisture assessment.

## 3. Materials and Methods

The experimental study included moisture-dependent measurements performed with patch antennas P1 and P2 and validation tests conducted with microstrip probe M1 using the PicoVNA and the developed TDR prototype. The materials, conditioning procedure, sensing elements, instrumentation, acquisition protocols, and data-processing methods are described in the following subsections.

### 3.1. Materials and Moisture-Conditioning Protocol

Three representative porous material configurations from the Apulia region were investigated: Lecce stone, Carparo, and plaster applied to a Carparo substrate. Lecce stone and Carparo are calcarenitic materials widely used in the historic built environment of Southern Italy, whereas the plaster-on-Carparo configuration was included to reproduce a layered surface condition commonly encountered in historic masonry. The Lecce stone and Carparo specimens had dimensions of 12 × 12 × 5 cm^3^, while the plaster-on-Carparo specimen had dimensions of 12 × 12 × 4 cm^3^. The latter consisted of a Carparo substrate covered by a single lime-based plaster layer approximately 3 mm thick. Carparo is characterized by a real density of 2.72 g/cm^3^, a dry bulk density of 1.58 g/cm^3^, and a total porosity of 41.9%, whereas Lecce stone has a real density of 2.72 g/cm^3^, an apparent density of 1.78 g/cm^3^, and a total porosity of 36.4%.

Carparo was selected for the TDR validation because, among the stone materials investigated in this study, it exhibited the highest total porosity and represents a material of particular relevance for historic and traditional masonry in Southern Italy, including structures affected by rising damp. Its use therefore provided a representative porous substrate for investigating moisture-induced electromagnetic variations during the saturation and drying process.

One specimen was considered for each material configuration. The same Carparo specimen was used in both the FDR and TDR experimental campaigns.

Before the electromagnetic measurements, each specimen was oven-dried at 105 °C for 24 h and subsequently allowed to cool before the oven-dry mass was recorded. The specimens were then fully immersed in water at room temperature for 24 h and subsequently monitored during natural evaporation. After immersion, they were removed from the water, and excess surface water was gently removed using absorbent paper before the first gravimetric and electromagnetic measurements were performed. The evaporation phase was carried out under laboratory conditions at approximately 22 + 2 °C and 50±10% relative humidity. At each selected measurement step, prior to gravimetric weighing and electromagnetic acquisition, the specimens were temporarily wrapped in plastic film to limit surface evaporation and promote moisture redistribution, thereby reducing moisture gradients within the specimens. The specimen mass and the corresponding electromagnetic response were then acquired at the same measurement step. Measurements were performed at intervals of approximately 30 min during the initial evaporation stage and at progressively longer intervals as the drying rate decreased. The monitoring procedure continued until the specimen mass approached the initial oven-dry value or no appreciable variation was observed between consecutive measurements. The same oven-drying, water-saturation, and natural-evaporation protocol was adopted for the Carparo specimen in both the FDR and TDR experiments.

**Figure 2:**
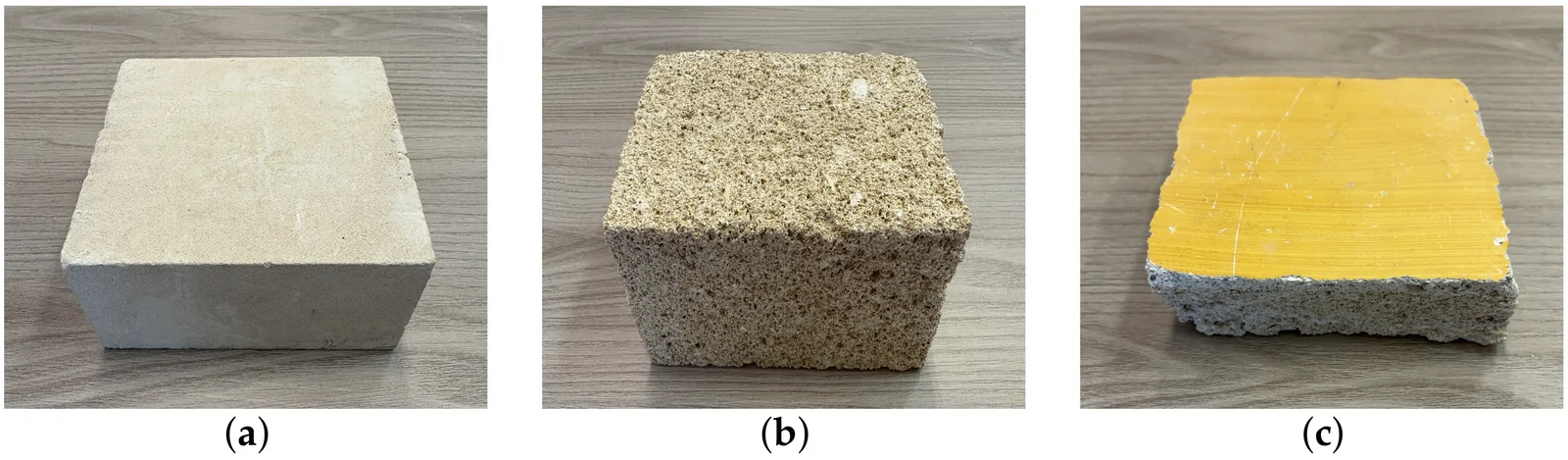
Investigated masonry materials: (**a**) Lecce stone, (**b**) Carparo, and (**c**) plaster applied to a Carparo substrate.

At the *i*th measurement step, the gravimetric moisture content was calculated with respect to the oven-dry mass as

(13) $$w_{i}=\frac{m_{i}-m_{\mathrm{dry}}}{m_{\mathrm{dry}}}\times 100\%,$$

where $m_{i}$ is the specimen mass measured at the *i*th step and $m_{\mathrm{dry}}$ is the corresponding oven-dry mass. The specimen mass was measured using an ORMA BC laboratory balance (ORMA S.r.l., Sesto San Giovanni, Milan, Italy) with a maximum capacity of 8000g and a resolution of 0.1g. Gravimetric weighing and electromagnetic acquisition were performed at the same moisture-conditioning steps so that each measured reflectometric response could be associated with the corresponding gravimetric moisture value [53]. The investigated masonry material configurations are shown in Figure 2.

### 3.2. Patch Antennas and FDR Measurement Setup

Two rectangular patch antennas, denoted as P1 and P2, were fabricated on an FR-4 substrate with thickness h=1.6mm and designed to operate at different resonance frequencies. The use of two resonant configurations was intended to investigate the moisture-dependent response under different operating frequencies and electromagnetic coupling conditions. The main nominal geometrical and electromagnetic parameters of the two antennas are summarized in Table 2.

**Figure 3:**
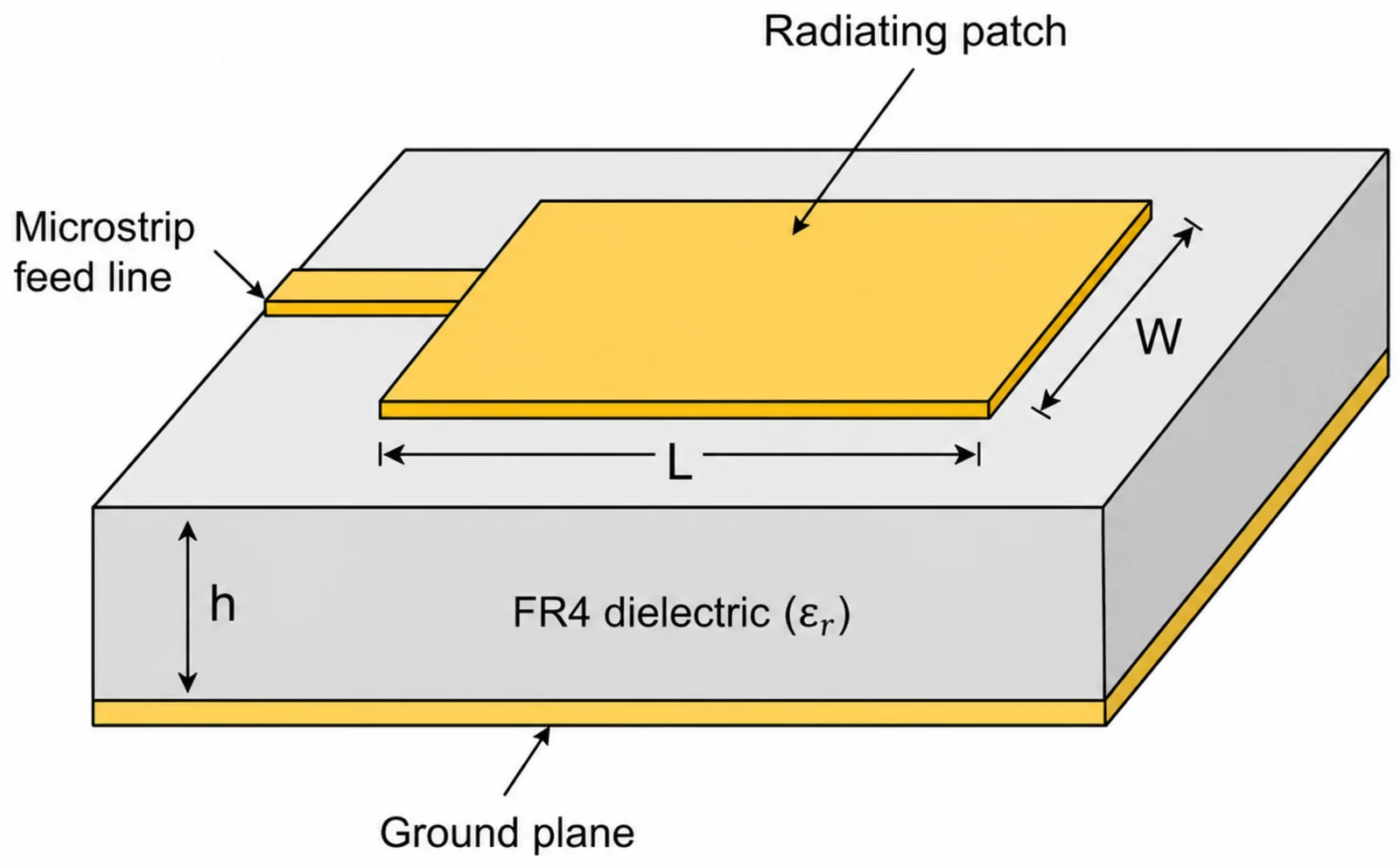
Schematic geometry of the rectangular patch antennas used as contact-based FDR sensors. The main geometrical parameters are the patch width *W*, patch length *L*, and substrate thickness *h*; the corresponding values for P1 and P2 are reported in Table 2.

The two antennas shared the same general layered architecture, consisting of a rectangular radiating patch and a microstrip feed line patterned on the upper surface of the FR-4 substrate, with a conductive ground plane on the opposite side. The main geometrical parameters are the patch width *W*, patch length *L*, and substrate thickness *h*, as defined in Figure 3. The corresponding values for P1 and P2 are reported in Table 2.

Before the moisture experiments, both antennas were modeled and simulated using CST Studio Suite 2019 (Dassault Systèmes Deutschland GmbH, Stuttgart, Germany) and subsequently characterized experimentally in air. The FR-4 substrate was represented using the lossy material model available in the CST material library, with relative permittivity $\varepsilon_{r}=4.3$, relative permeability $\mu_{r}=1$, and dielectric loss tangent tanδ=0.025. This preliminary characterization was performed to verify the position, depth, and overall shape of the principal resonance and to assess the consistency between the numerical model and the fabricated antennas.

**Figure 4:**
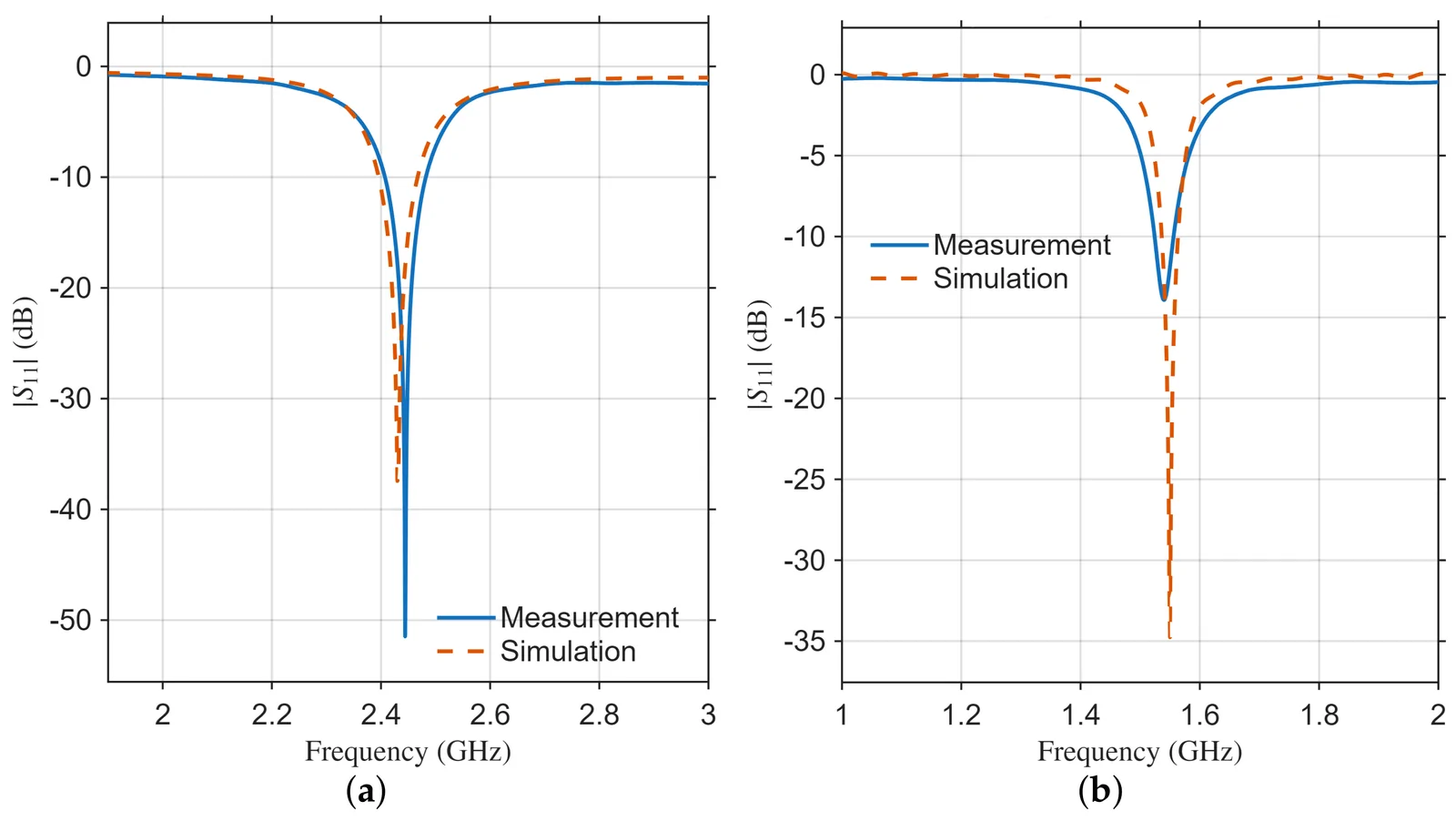
Comparison between the CST-simulated and experimentally measured $|S_{11}\left(f\right)|$ responses in air for (**a**) antenna P1 and (**b**) antenna P2.

The simulated and measured responses exhibited comparable resonant behavior, as shown in Figure 4. The remaining differences may be attributed to fabrication tolerances, deviations between the nominal and actual dielectric properties of the FR-4 substrate, uncertainty in conductor and substrate dimensions, connector and feeding effects, and imperfections in the experimental measurement path.

Prior to the moisture-dependent experiments, the influence of specimen thickness on the sensor response was preliminarily assessed under dry conditions using specimens with progressively increasing thicknesses (approximately 1–5 cm). The measured $S_{11}$ response progressively stabilized as the specimen thickness increased, with no appreciable variations in resonance position or overall spectral behavior beyond approximately 2 cm for P1 and 3 cm for P2. These observations indicated that the specimen thicknesses adopted in the subsequent experiments were sufficient to minimize the influence of the rear sample boundary. The different stabilization thicknesses are qualitatively consistent with the different operating frequencies of the two sensing structures, with the lower-frequency P2 configuration expected to interact with a comparatively larger material volume than the higher-frequency P1 configuration. For the contact-based resonant structures considered here, however, the effective sensing depth does not correspond to a sharply defined physical boundary, but depends jointly on operating frequency, sensor geometry, and the electromagnetic properties of the investigated material.

The FDR measurements were performed using a PicoVNA 108 VNA (Pico Technology Ltd., St Neots, Cambridgeshire, UK) connected to the selected patch antenna through a coaxial cable. The instrument operates over a nominal frequency range from 300kHz to 8.5GHz and was controlled through a Microsoft Windows 11 computer via Universal Serial Bus (USB) using the PicoVNA software version 5. The analyzer supports complex scattering-parameter acquisition, reference-plane offset and de-embedding, time-domain transformations, and data export in tabular, graphical, and Touchstone formats. Its compact and lightweight architecture makes it suitable for transportable laboratory measurements, although the present experimental campaign was conducted under controlled laboratory conditions.

Only one-port reflection measurements were required in the present study. Before each measurement session, a Short–Open–Load (SOL) calibration was performed using the original Pico Technology calibration kit supplied with the PicoVNA 108. For the one-port measurements, the short, open, and 50Ω load standards were sequentially connected at the end of the measurement cable. The calibration reference plane was therefore placed at the cable connector subsequently connected to the patch antenna. This procedure compensated for systematic contributions associated with directivity, source match, and the frequency response of the coaxial cable and connection path.

**Figure 5:**
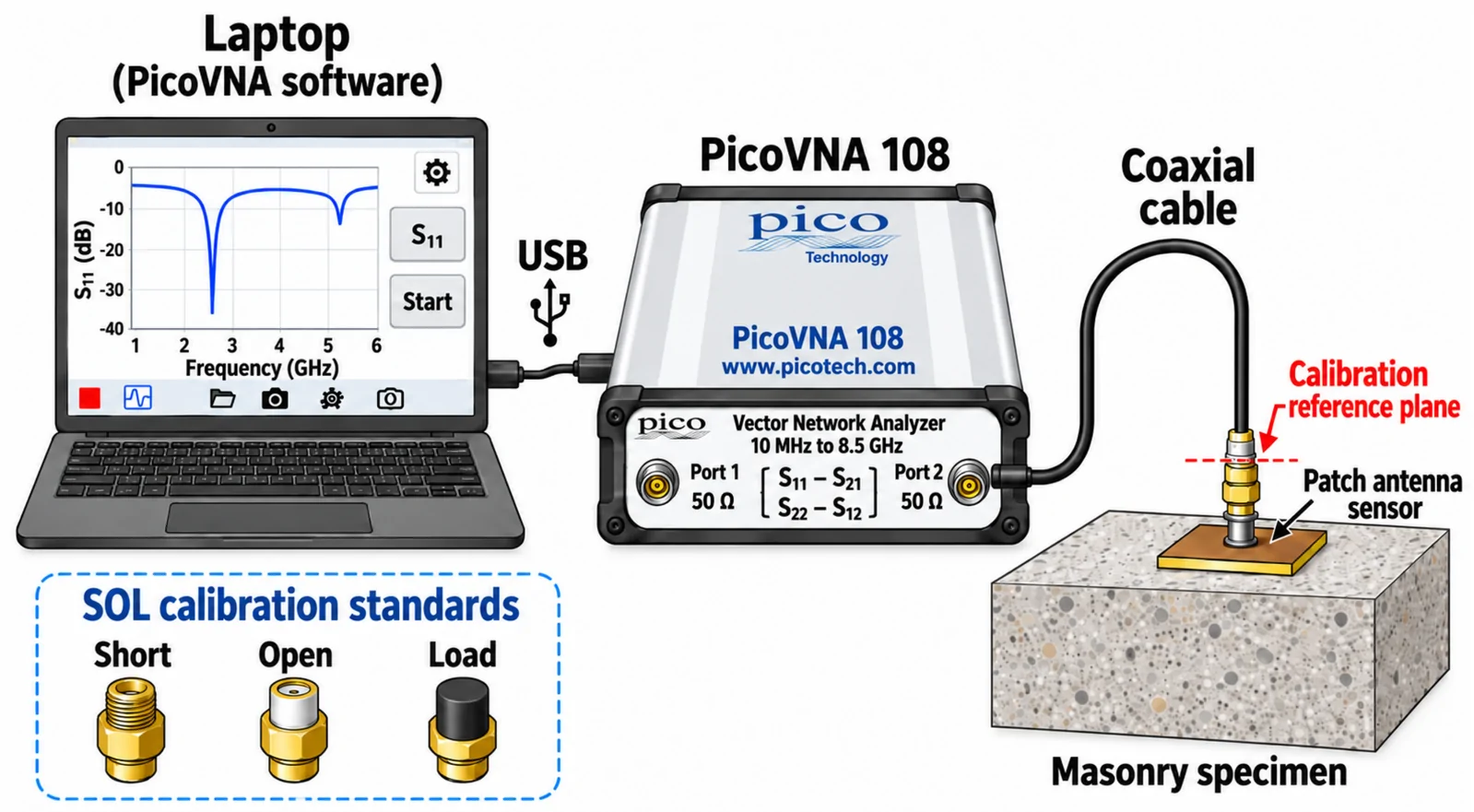
Schematic representation of the FDR measurement setup. The PicoVNA 108 was controlled through a laptop and connected to the contact-based patch antenna by means of a coaxial cable. A Short–Open–Load calibration was performed at the cable-end reference plane before connecting the antenna and placing it directly on the masonry specimen.

After calibration, the antenna was connected without modifying the cable configuration and was placed directly on the specimen surface, with the radiating patch facing the material. The antenna was not bonded to the specimen and no intermediate coupling layer was used; it was carefully positioned in direct contact with the surface so as to maximize the contact area and minimize possible air gaps at the antenna–material interface. The same nominal position and orientation were maintained throughout the drying sequence to reduce variability associated with repositioning and contact conditions and to ensure consistency among measurements performed at different moisture levels. The FDR measurement arrangement, including the PicoVNA 108, the SOL calibration standards, the cable-end reference plane, and the contact configuration between the patch antenna and the masonry specimen, is shown in Figure 5.

At each moisture level, the complex reflection coefficient $S_{11}\left(f\right)$ was acquired over a frequency interval centered on the principal resonance of the selected antenna. To improve measurement quality and reduce random fluctuations, the response associated with each moisture-conditioning step was obtained by averaging 64 consecutive acquisitions. The resonance frequency $f_{\mathrm{res}}$ was identified as the frequency corresponding to the minimum of $|S_{11}\left(f\right)|$ within a predefined frequency interval containing the principal resonance. The extracted resonance frequencies were subsequently related to the corresponding gravimetric moisture values to derive a separate calibration relationship for each sensor–material combination.

### 3.3. Microstrip Probe and Portable TDR Prototype

The TDR validation experiments were performed using a dedicated planar microstrip probe, hereafter denoted as M1, fabricated on an FR-4 substrate. As aforementioned, owing to its elongated and scalable geometry, M1 is particularly suitable for applications involving rising damp, since the sensing structure can be extended along a wall section to investigate moisture variations over a broader vertical region than a localized patch antenna. The probe consisted of a straight sensing conductor patterned on the upper surface of the substrate and a continuous conductive ground plane on the opposite side. Its geometry was selected so that the principal resonance fell within the usable bandwidth of the portable TDR prototype and could therefore be recovered after time-to-frequency-domain reconstruction.

**Figure 6:**
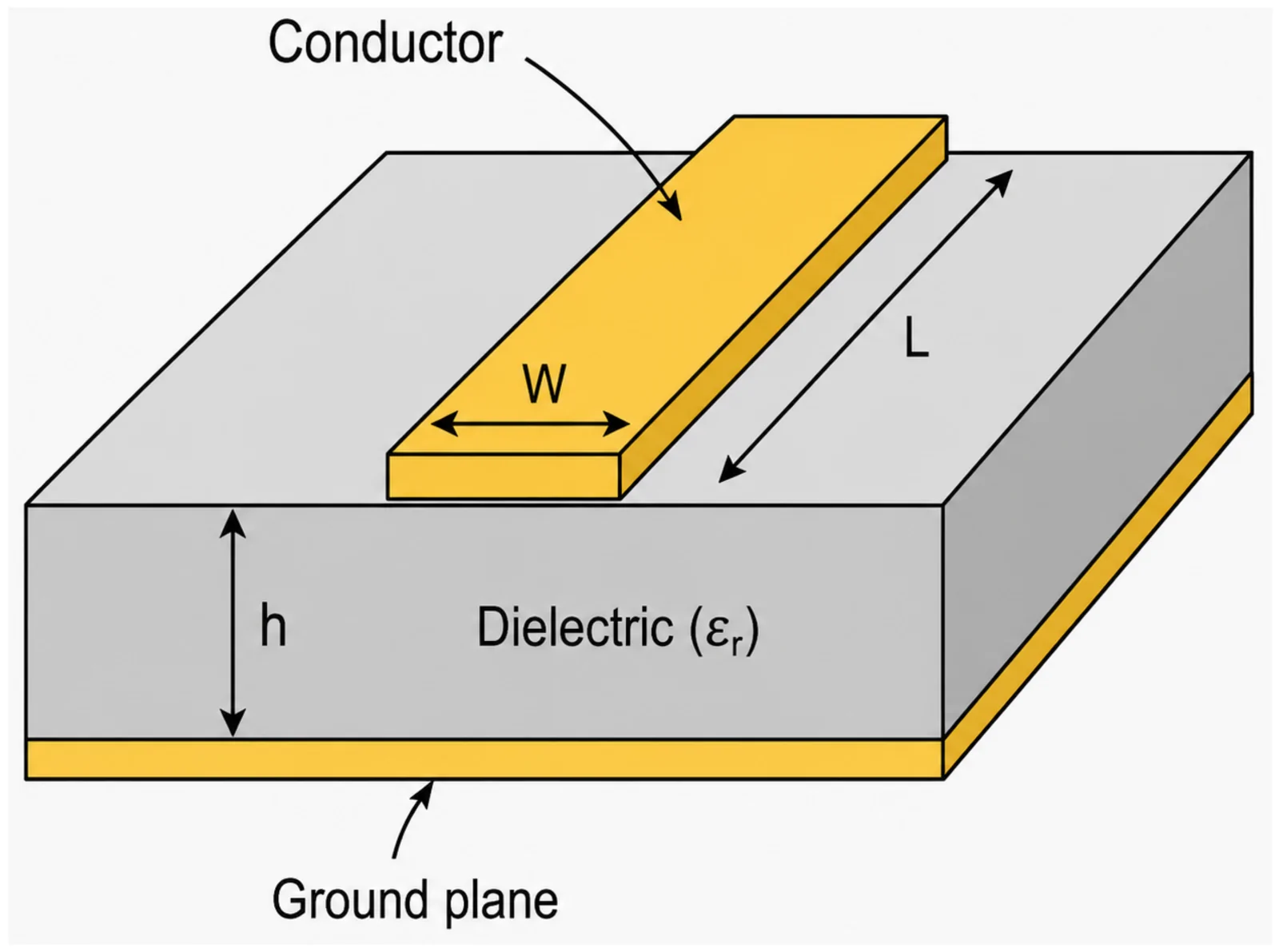
Schematic geometry of microstrip probe M1. The conductor width *W*, effective conductor length *L*, and substrate thickness *h* are defined in the schematic and reported in Table 3.

The sensing conductor had a nominal width W=2mm and an effective length L=101.5mm, while the FR-4 substrate had a thickness h=1.6mm. The overall fabricated length was approximately 113mm, including the feeding and connection regions not included in the effective sensing length *L*. Under free-space conditions, M1 exhibited a principal resonance at approximately 852.22MHz. The main nominal parameters are summarized in Table 3. The geometry of M1 and the corresponding geometrical parameters are illustrated in Figure 6.

The approximately 11-cm overall length of M1 was selected in accordance with the specific application scenario considered in this study and with the usable bandwidth of the portable TDR prototype. In particular, the elongated geometry is intended for monitoring extended masonry regions, such as wall sections affected by rising damp, rather than for localized measurements on small surface areas. At the selected resonance frequency of approximately 852 MHz, a conventional patch antenna would require a comparatively larger surface area, whereas the elongated microstrip configuration provides a more suitable geometry for the investigated specimens and the intended monitoring application. For applications involving irregular, curved, or geometrically constrained masonry surfaces, the sensing geometry can instead be specifically adapted to the available contact area, for example by reducing the probe dimensions or by adopting alternative planar configurations.

A similar preliminary thickness-sensitivity assessment was performed for M1 under dry conditions. No appreciable changes in the measured response were observed for specimen thicknesses greater than approximately 4 cm, indicating that the adopted Carparo specimen thickness was sufficient to minimize rear-boundary effects.

**Figure 7:**
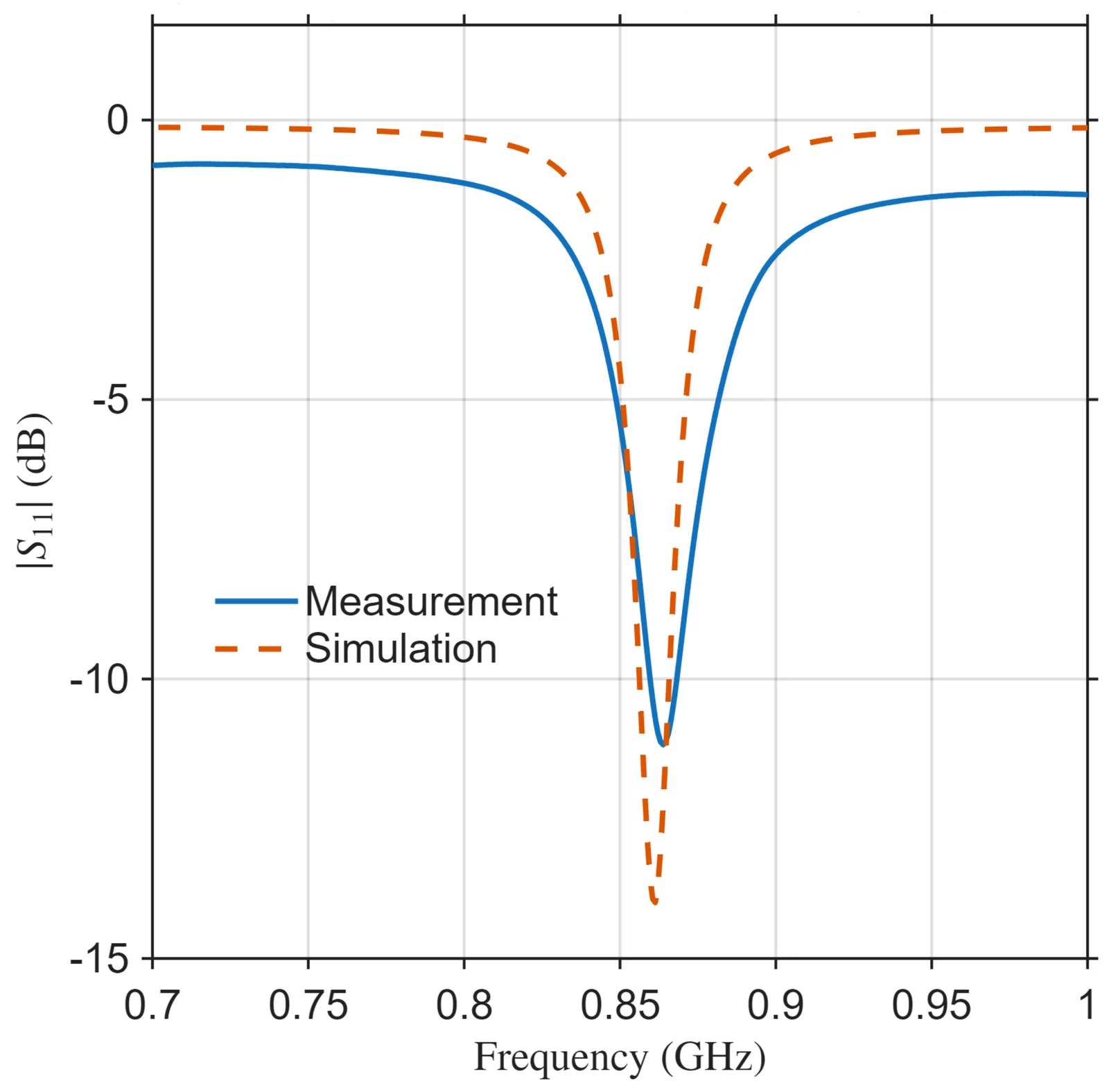
Comparison between the CST-simulated and experimentally measured $|S_{11}\left(f\right)|$ responses in air for microstrip probe M1.

As for P1 and P2, the electromagnetic design of M1 was assessed by comparing the CST-simulated and experimentally measured $|S_{11}\left(f\right)|$ responses in air, as shown in Figure 7. This comparison was used to verify the position and overall shape of the principal resonance before the moisture experiments.

**Figure 8:**
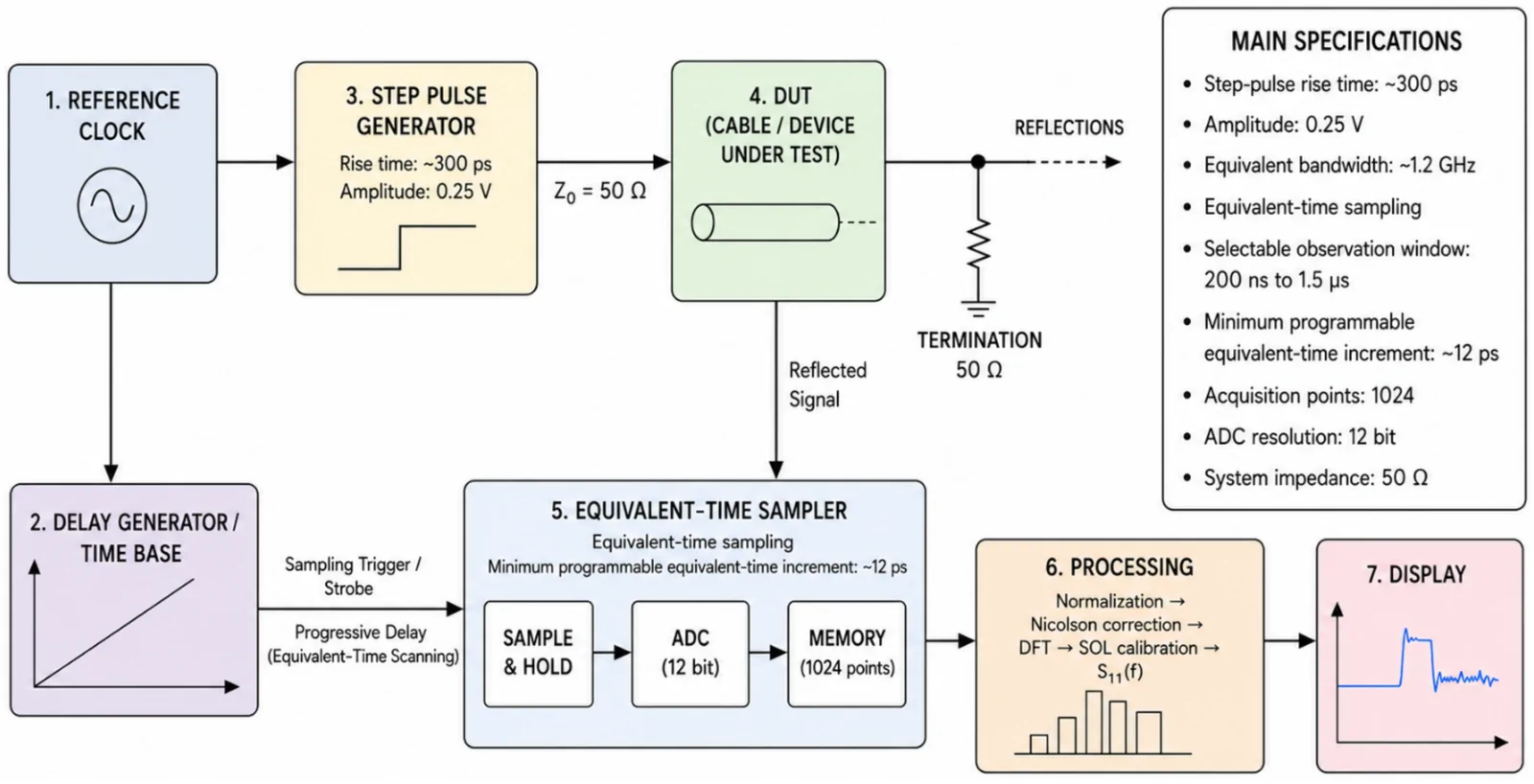
Block diagram of the developed portable TDR prototype. The system comprises a reference clock, a delay generator/time-base, a step-pulse generator, the device-under-test (DUT) measurement path, an equivalent-time sampling stage including sample-and-hold, 12-bit ADC, and 1024-point memory, followed by digital processing and display. The principal operating specifications of the prototype are summarized in the figure. The arrows indicate the direction of signal generation, propagation, acquisition, and subsequent processing through the measurement chain.

The time-domain measurements were acquired using a custom portable TDR prototype developed within the authors’ research activities. The system was assembled using commercially available components and a simplified hardware architecture, with an estimated hardware cost of approximately EUR 500. As illustrated in Figure 8, the prototype comprises a reference clock, a delay-generator/time-base unit, a fast step-pulse generator, the measurement path connected to the device under test (DUT), an equivalent-time sampling and acquisition stage, and digital processing and display sections. Dedicated software is used for instrument control, waveform acquisition, data storage, signal export, and time-to-frequency-domain processing.

The generated step signal has a nominal rise time of approximately 300ps and an amplitude of 0.25 V, corresponding to an equivalent bandwidth of approximately 1.2GHz. The measurement path has a nominal characteristic impedance of 50 Ω. Signal acquisition is performed using equivalent-time sampling, in which a progressively delayed sampling instant is used over successive acquisitions to reconstruct the time-domain waveform. The prototype provides a selectable observation window ranging from 200ns to 1.5 μs, with 1024 acquisition points, a 12-bit analog-to-digital converter (ADC), and a minimum programmable equivalent-time increment of approximately 12ps. The available bandwidth includes the free-space resonance of M1 and therefore permits recovery of its principal spectral feature from the measured time-domain response.

Following acquisition, the reconstructed reflectograms are processed through normalization, Nicolson correction, discrete Fourier transformation (DFT), and one-port Short–Open–Load (SOL) calibration, as detailed in Section 2.3, to obtain the corresponding complex frequency-domain reflection coefficient $S_{11}\left(f\right)$.

The prototype architecture was conceived to support portable operation and possible future integration into remotely controlled or distributed monitoring systems. Nevertheless, the measurements presented in this study were performed under controlled laboratory conditions, and no field or remote-monitoring validation was carried out.

For the validation campaign, M1 was placed in contact with the selected Carparo specimen and connected alternately to the PicoVNA 108 and to the portable TDR prototype. Measurements with both instruments were performed at the same gravimetric moisture levels during natural evaporation. The probe position, orientation, and nominal contact configuration were maintained unchanged as far as practicable to limit differences associated with coupling and repositioning.

The PicoVNA provided both the directly measured complex frequency-domain response $S_{11}\left(f\right)$ and a corresponding time-domain representation obtained through its time-domain function. The portable prototype directly acquired the reflectograms. For each moisture-conditioning step, 128 consecutive reflectograms were averaged before the subsequent processing in order to improve the signal-to-noise ratio and reduce random acquisition fluctuations. The averaged reflectograms were subsequently processed using the reconstruction procedure described in Section 2.3. This arrangement enabled two complementary comparisons: a time-domain comparison between the PicoVNA and prototype responses, and a frequency-domain comparison between the directly measured and TDR-reconstructed $S_{11}\left(f\right)$ curves.

### 3.4. Experimental Workflow

**Figure 9:**
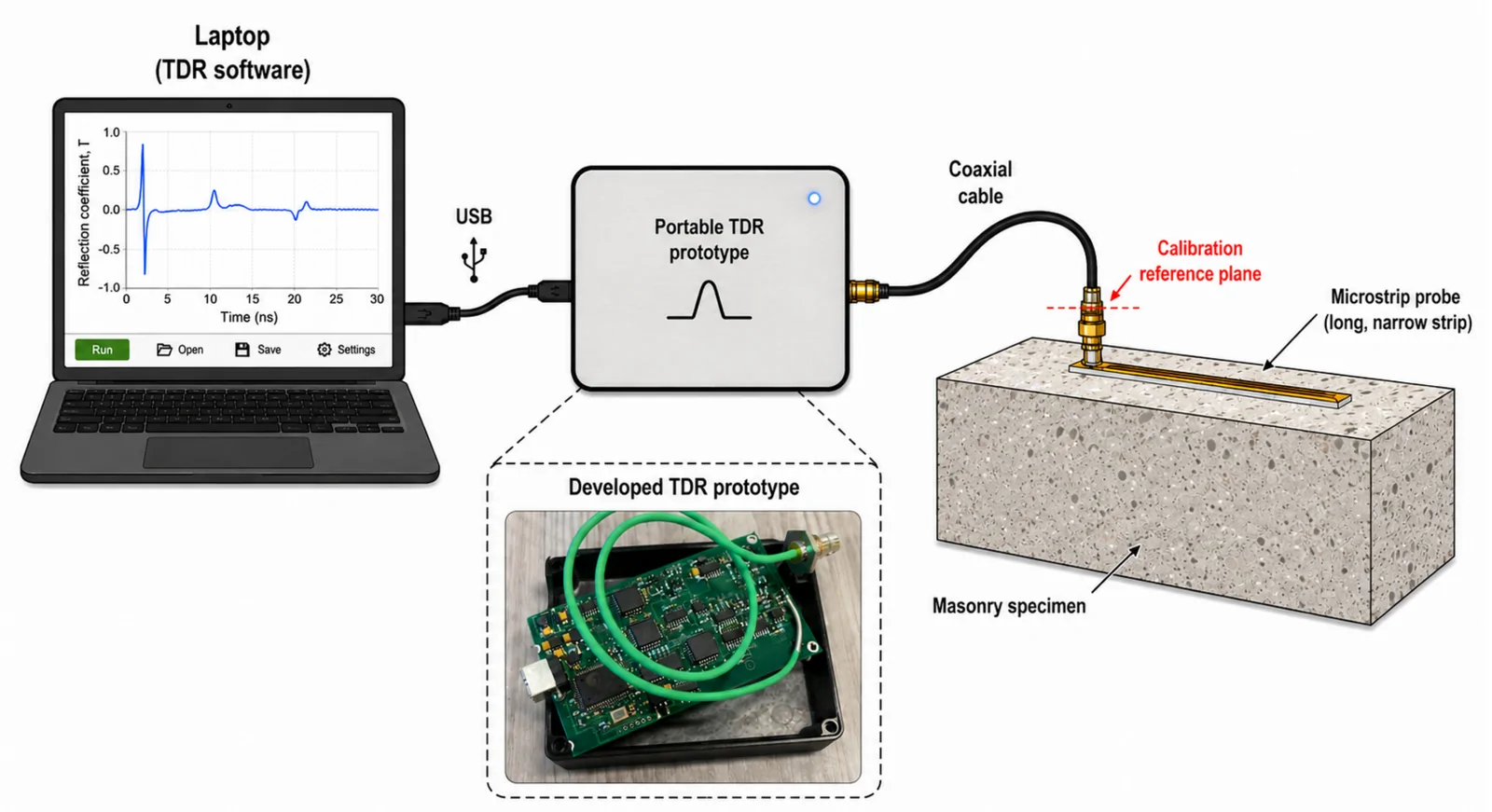
Portable TDR measurement setup comprising the control laptop, developed TDR prototype, coaxial connection, and planar microstrip probe M1 placed in contact with the masonry specimen. The calibration reference plane is indicated at the cable-to-probe connection, while the inset shows the prototype internal electronics.

The experimental workflow was organized into two connected branches: a VNA-based frequency-domain characterization using the patch antennas P1 and P2, and the validation of the developed portable TDR prototype using the microstrip probe M1. In both branches, the electromagnetic measurements were associated with gravimetric moisture values determined during the controlled drying of the masonry specimens. At each acquisition step, the specimen mass was recorded and the corresponding gravimetric moisture content was calculated according to the procedure described in Section 3.1. The portable TDR measurement setup used for the validation experiments is shown in Figure 9.

In the FDR branch, patch antennas P1 and P2 were used with the PicoVNA 108 to characterize Lecce stone, Carparo, and plaster on Carparo. For each antenna, the frequency-domain response was acquired at successive gravimetric moisture levels while maintaining the sensor position and contact configuration as consistently as practicable. This procedure produced six material–antenna datasets, corresponding to P1 and P2 applied separately to the three masonry configurations. The resonance frequency extracted from each acquisition was subsequently associated with the gravimetric moisture content measured at the same drying step.

The second branch was devoted to the validation of the portable TDR prototype using the microstrip probe M1 on Carparo. The validation comprised a preliminary free-space comparison and a subsequent moisture-dependent investigation on the masonry specimen.

Under free-space conditions, M1 was characterized using both measurement chains. The time-domain response obtained using the PicoVNA in TDR mode was compared with the reflectogram acquired directly using the portable prototype. The prototype reflectogram was then processed using the time-to-frequency-domain reconstruction procedure described in Section 2.3, and the resulting frequency response was compared with that measured directly using the PicoVNA.

The moisture-dependent validation was subsequently performed during controlled drying of the Carparo specimen. At each selected gravimetric moisture level, four corresponding responses were obtained:

1. The frequency-domain response measured directly using the PicoVNA 108;
2. The time-domain response obtained using the PicoVNA in TDR mode;
3. The reflectogram acquired directly using the portable TDR prototype;
4. The frequency-domain response reconstructed from the prototype reflectogram.

These acquisitions enabled two complementary comparisons. In the time domain, the moisture-dependent reflectograms obtained using the PicoVNA and the portable prototype were examined in terms of their apparent-distance evolution and response ordering. In the frequency domain, the responses reconstructed from the prototype measurements were compared with those measured directly using the PicoVNA over their common usable frequency range.

For each moisture level, the resonance frequency of M1 was extracted independently from the PicoVNA-measured and TDR-reconstructed responses. The paired resonance-frequency values were then used to quantify the agreement between the two measurement chains according to the procedure described in Section 3.5.

**Figure 10:**
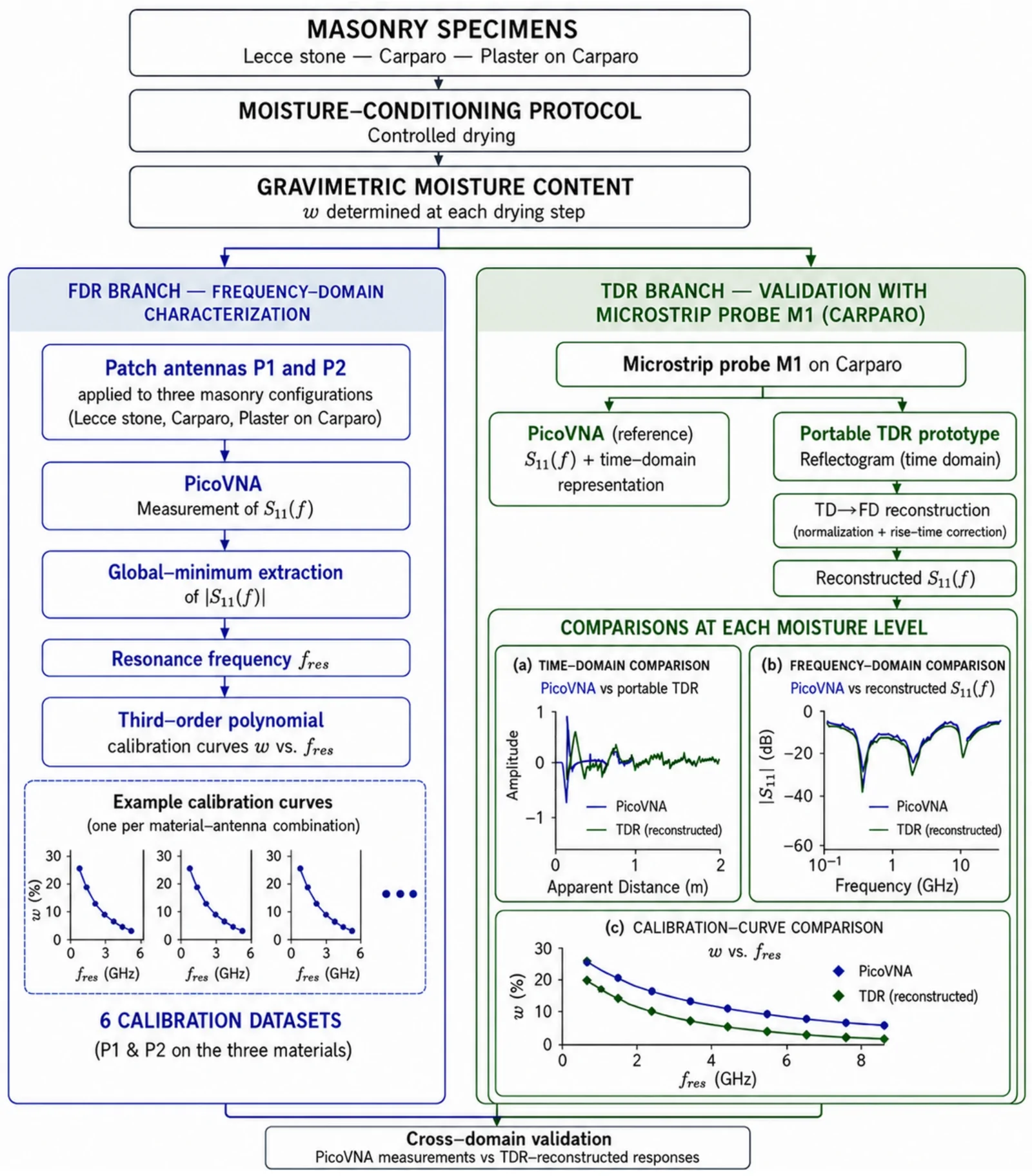
Overview of the experimental workflow adopted for the VNA-based frequency-domain characterization and the validation of the portable TDR prototype.

The complete workflow therefore comprised: (i) multi-material FDR characterization using P1 and P2; (ii) free-space validation of M1 in the time and frequency domains; (iii) moisture-dependent comparison of the PicoVNA and portable-TDR responses on Carparo; and (iv) quantitative comparison of the resonance frequencies obtained from the two measurement chains. A schematic overview is provided in Figure 10.

### 3.5. Feature Extraction, Calibration, and Cross-Domain Comparison

The acquired reflectometric responses were processed using procedures specific to the FDR characterization and TDR-validation branches. For the FDR datasets, the analysis focused on resonance-frequency extraction and gravimetric calibration. For the TDR branch, the comparison was performed in both the time and frequency domains and was completed by a quantitative evaluation of the resonance-frequency differences between the two measurement chains.

For the FDR measurements, the resonance frequency of each patch antenna was selected as the primary moisture-sensitive feature. For every combination of masonry material, patch antenna, and moisture-conditioning step, $f_{\mathrm{res}}$ was identified as the frequency corresponding to the minimum of the measured $|S_{11}\left(f\right)|$ response within a predefined frequency interval containing the principal resonance. The extracted resonance frequency was paired with the gravimetric moisture content measured at the same experimental step.

Separate calibration datasets were obtained for each combination of the three masonry configurations and the two patch antennas, resulting in six material–antenna datasets. For each antenna, ten measurement points were available for Lecce stone, eight for Carparo, and seven for plaster on Carparo. More generally, recent TDR studies on porous building materials have highlighted the importance of calibration-model selection and data-processing strategies for improving moisture-estimation accuracy [54,55].

In this context, a third-order polynomial regression was fitted independently to each dataset to describe the nonlinear relationship between resonance frequency and gravimetric moisture content:

(14) $$w=a_{3}f_{\mathrm{res}}^{3}+a_{2}f_{\mathrm{res}}^{2}+a_{1}f_{\mathrm{res}}+a_{0},$$

where $f_{\mathrm{res}}$ is expressed in GHz, *w* is the gravimetric moisture content expressed as a percentage, and $a_{0}$, $a_{1}$, $a_{2}$, and $a_{3}$ are the regression coefficients estimated separately for each material–antenna combination. The same polynomial order was adopted for all six datasets to provide a consistent representation of the observed nonlinear calibration trends.

The goodness of fit was quantified using the coefficient of determination,

(15) $$R^{2}=1-\frac{\sum_{i=1}^{n}{\left(w_{i}-{\hat{w}}_{i}\right)}^{2}}{\sum_{i=1}^{n}{\left(w_{i}-\bar{w}\right)}^{2}},$$

where $w_{i}$ is the gravimetric moisture content measured at the *i*th experimental step, ${\hat{w}}_{i}$ is the corresponding value predicted by the fitted calibration curve, $\bar{w}$ is the mean gravimetric moisture content, and *n* is the number of measurement points. The fitted coefficients and corresponding $R^{2}$ values are reported with the FDR results.

For the TDR branch, the comparison was carried out separately in the time and frequency domains. In the time domain, the moisture-dependent responses obtained using the PicoVNA in TDR mode were compared with the reflectograms directly acquired using the portable prototype. The analysis considered the apparent position of the principal response features, their overall waveform evolution, and the ordering of the traces across the investigated gravimetric moisture levels.

The prototype reflectograms were subsequently processed according to Section 2.3. After baseline normalization, Nicolson correction, discrete Fourier transformation, and one-port calibration, the reconstructed complex $S_{11}\left(f\right)$ responses were compared with those measured directly using the PicoVNA over the common usable frequency range. For each moisture level, the resonance frequency was identified as the minimum of the corresponding $|S_{11}\left(f\right)|$ response within the predefined frequency interval containing the principal resonance of M1.

The resonance-frequency difference at the *i*th moisture level was defined as

(16) $$\Delta f_{\mathrm{res},i}=f_{\mathrm{res},i}^{\mathrm{TDR}}-f_{\mathrm{res},i}^{\mathrm{VNA}},$$

where $f_{\mathrm{res},i}^{\mathrm{TDR}}$ and $f_{\mathrm{res},i}^{\mathrm{VNA}}$ are the resonance frequencies obtained from the TDR-reconstructed and PicoVNA-measured responses, respectively.

The agreement between the two measurement chains was summarized using the mean resonance-frequency bias,

(17) $${\bar{\Delta f}}_{\mathrm{res}}=\frac{1}{n}\sum_{i=1}^{n}\Delta f_{\mathrm{res},i},$$

the standard deviation SD of the differences,

(18) $$s_{\Delta f}=\sqrt{\frac{1}{n-1}\sum_{i=1}^{n}{\left(\Delta f_{\mathrm{res},i}-{\bar{\Delta f}}_{\mathrm{res}}\right)}^{2}},$$

and the mean absolute error MAE and root mean square error RMSE.

(19) $${\mathrm{MAE}}_{f}=\frac{1}{n}\sum_{i=1}^{n}\left|f_{\mathrm{res},i}^{\mathrm{TDR}}-f_{\mathrm{res},i}^{\mathrm{VNA}}\right|,$$

(20) $${\mathrm{RMSE}}_{f}=\sqrt{\frac{1}{n}\sum_{i=1}^{n}{\left(f_{\mathrm{res},i}^{\mathrm{TDR}}-f_{\mathrm{res},i}^{\mathrm{VNA}}\right)}^{2}}.$$

These indicators were calculated over all moisture levels investigated for M1 on Carparo. The mean bias was used to identify any systematic frequency offset between the two measurement chains, while the standard deviation quantified the variability of this offset across the drying process. The MAE and RMSE described the overall magnitude of the resonance-frequency discrepancies, with the RMSE assigning greater weight to larger deviations.

The resulting metrics were interpreted as indicators of consistency between the PicoVNA-measured and TDR-reconstructed responses under the investigated laboratory conditions. They were not intended to establish full metrological equivalence, which would require repeated independent acquisitions, a dedicated uncertainty budget, and validation across additional probes, masonry materials, and environmental conditions.

## 4. Results

### 4.1. FDR-Based Moisture Characterization Using Patch Antennas

This subsection presents the moisture-dependent frequency-domain results obtained using patch antennas P1 and P2 on Lecce stone, Carparo, and plaster on Carparo. The analysis is first focused on the evolution of the measured $|S_{11}\left(f\right)|$ responses during drying and subsequently on the resonance-frequency calibration relationships obtained for each material–antenna combination.

#### 4.1.1. Moisture-Dependent Frequency Responses

**Figure 11:**
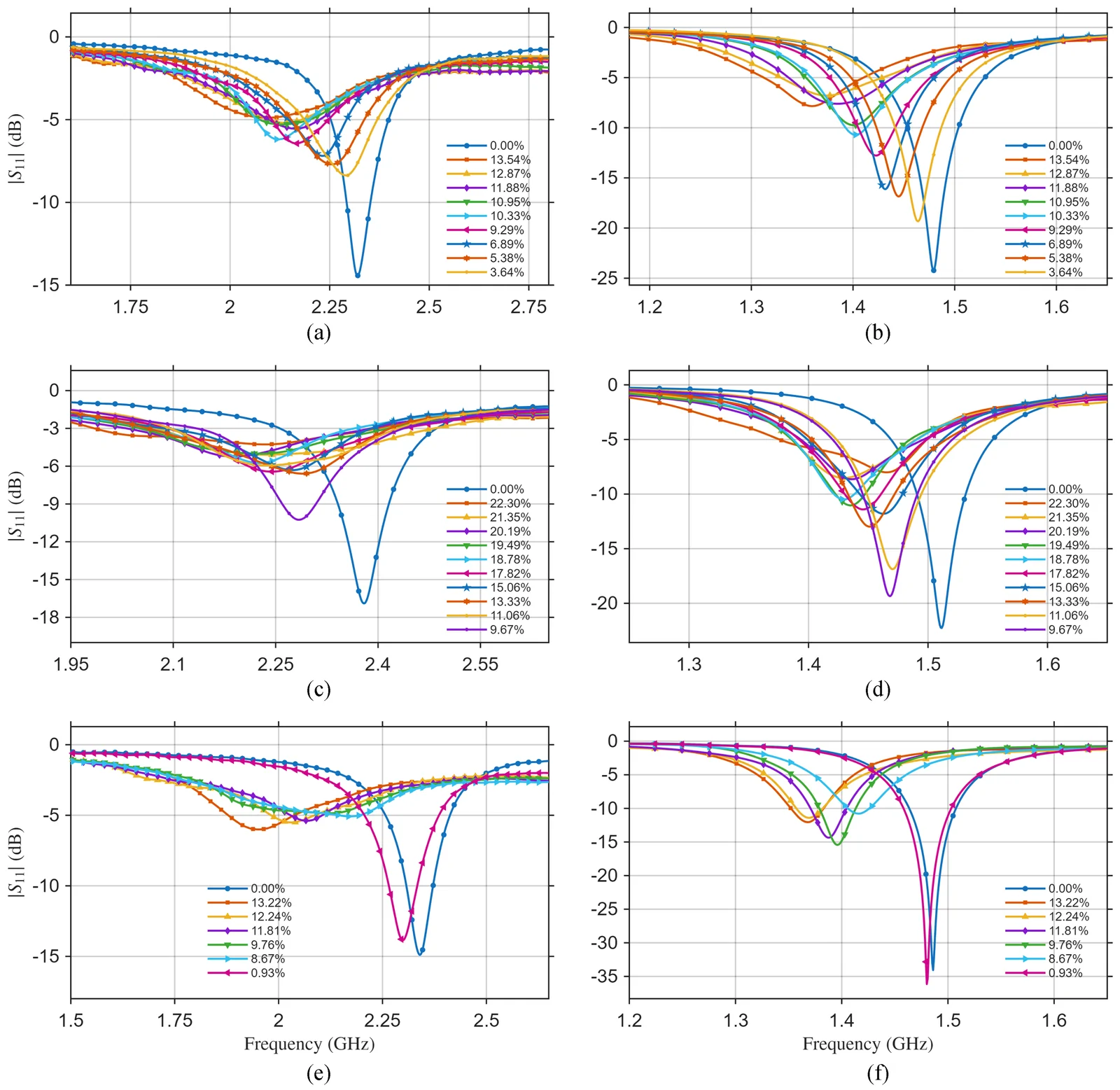
Moisture-dependent FDR responses measured using patch antennas P1 and P2: (**a**) Lecce stone–P1; (**b**) Lecce stone–P2; (**c**) Carparo–P1; (**d**) Carparo–P2; (**e**) plaster on Carparo–P1; and (**f**) plaster on Carparo–P2. Each panel shows the evolution of the measured $|S_{11}\left(f\right)|$ response at successive gravimetric moisture levels during controlled drying.

The frequency-domain measurements were examined to assess the evolution of the resonant response of patch antennas P1 and P2 during controlled drying. Figure 11 presents the six material–antenna datasets, with each curve associated with the gravimetric moisture content determined at the corresponding acquisition step.

A clear and progressive displacement of the principal resonance is visible for both antennas on all three masonry configurations. As the specimens dried, the resonance generally moved toward higher frequencies, in agreement with the reduction in the effective dielectric permittivity produced by the decreasing water content. The dry-state response was clearly distinguishable from the moisture-conditioned responses, while the intermediate curves described the gradual evolution of the sensor response throughout the drying process. The two antennas provided complementary frequency-domain characterizations. P1 operated within the higher-frequency range and exhibited a readily identifiable moisture-dependent resonance shift for all three configurations. P2 showed the same overall trend at its lower operating frequency, generally producing pronounced resonance minima and a clear separation among successive moisture conditions. The consistency of the shift observed at two different operating frequencies confirms the robustness of resonance-based moisture sensing and highlights the flexibility offered by antennas designed for different spectral ranges. Differences in resonance position, depth, and shape were observed among Lecce stone, Carparo, and plaster on Carparo, reflecting the different electromagnetic response of each masonry configuration. Accordingly, the resonance-frequency shift was calibrated separately for each sensor–material combination, rather than using a single relationship for all configurations. The material is therefore assumed to be known a priori, and the observed differences among the responses should not be interpreted as a material-identification capability of the proposed approach. The resulting calibration curves were then used to relate the measured resonance frequency to gravimetric moisture content.

Overall, the six datasets provide a coherent description of the moisture-dependent spectral evolution of the investigated specimens. The resonance frequency could be consistently identified from the minimum of each $|S_{11}\left(f\right)|$ response within the predefined frequency interval containing the principal resonance and was therefore selected as the calibration feature for the subsequent quantitative analysis.

#### 4.1.2. Resonance-Based Calibration Curves

The resonance frequencies extracted from the moisture-dependent $|S_{11}\left(f\right)|$ responses were associated with the corresponding gravimetric moisture contents measured at the same drying steps. Separate calibration relationships were obtained for P1 and P2 on Lecce stone, Carparo, and plaster on Carparo, resulting in six material–antenna datasets. A third-order polynomial model was fitted independently to each dataset, as described in Section 3.5.

**Figure 12:**
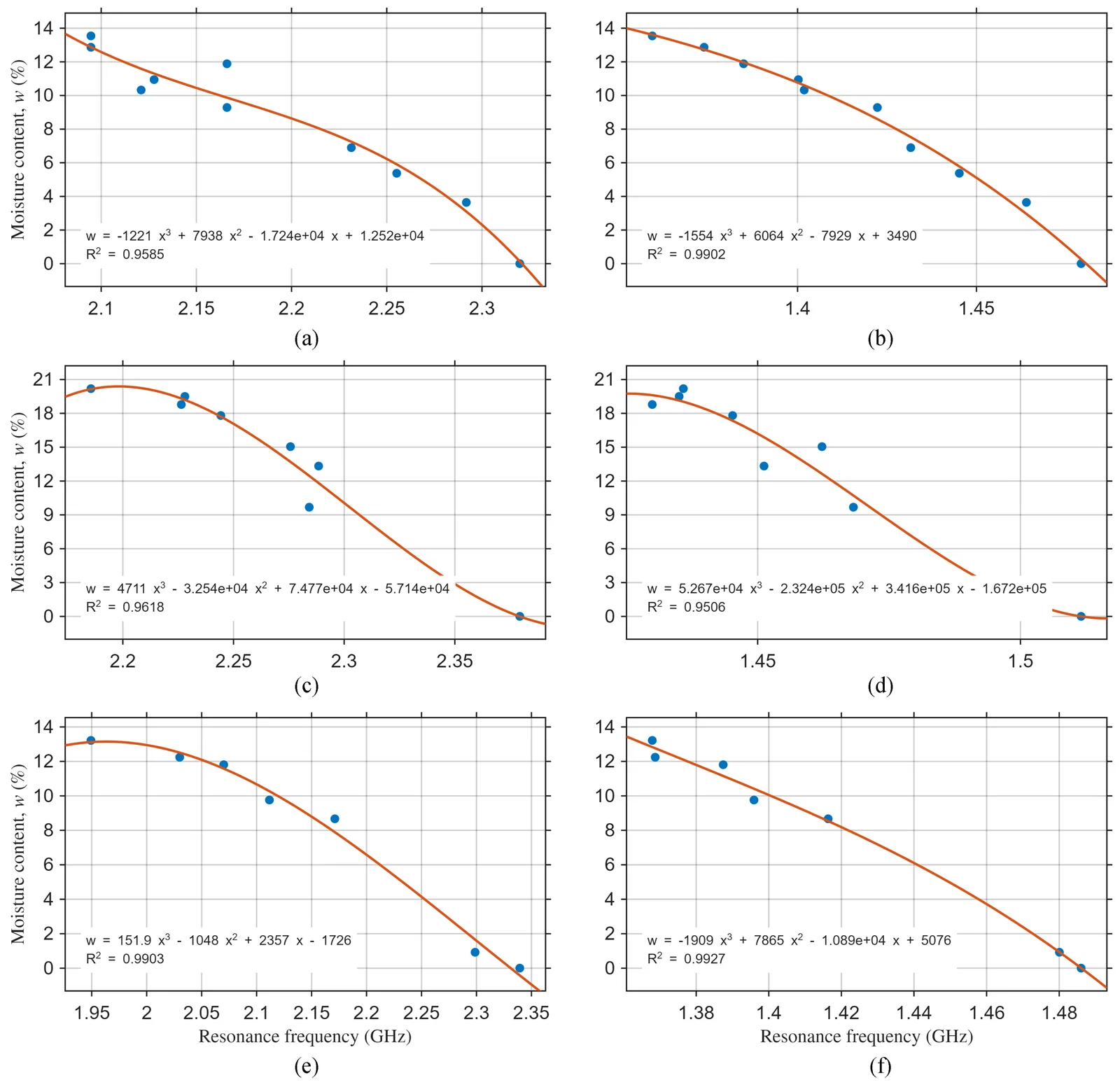
Gravimetric moisture–resonance frequency calibration curves obtained using patch antennas P1 and P2: (**a**) Lecce stone–P1; (**b**) Lecce stone–P2; (**c**) Carparo–P1; (**d**) Carparo–P2; (**e**) plaster on Carparo–P1; and (**f**) plaster on Carparo–P2. Experimental data points are shown together with the corresponding third-order polynomial fits. The fitted equation and coefficient of determination, $R^{2}$, are reported in each panel.

Figure 12 shows that the resonance-frequency shift observed during drying can be represented by well-defined nonlinear relationships for all six material–antenna combinations. In each case, increasing resonance frequency corresponds to decreasing gravimetric moisture content, consistently with the reduction in effective dielectric permittivity as water is progressively removed from the porous structure.

The calibration behavior remained material- and antenna-specific. Lecce stone exhibited a smooth decrease in gravimetric moisture with increasing resonance frequency for both P1 and P2. The corresponding coefficients of determination were $R^{2}=0.9585$ and $R^{2}=0.9902$, respectively, indicating that both antennas captured the moisture-dependent trend, with P2 providing the closest fit for this material. For Carparo, the calibration curves showed a more pronounced nonlinear profile over the investigated moisture range. The models obtained with P1 and P2 achieved $R^{2}=0.9618$ and $R^{2}=0.9506$, respectively. These values confirm that the resonance frequency remained strongly related to gravimetric moisture even for this highly porous calcarenitic material and across the two different operating-frequency ranges. The plaster-on-Carparo configuration provided the highest overall fitting coefficients. The calibration models reached $R^{2}=0.9903$ for P1 and $R^{2}=0.9927$ for P2, demonstrating a close correspondence between the extracted resonance frequencies and the gravimetric moisture values throughout the controlled drying process. The consistency obtained with both antennas confirms the capability of the proposed contact-based sensing approach to characterize moisture variations in a layered masonry configuration. The fitted coefficients and coefficients of determination are summarized in Table 4.

Overall, all six polynomial models achieved $R^{2}$ values greater than 0.95, supporting the use of resonance frequency as a robust moisture-sensitive feature within the investigated laboratory conditions. The results also confirm that sensors operating at different resonance frequencies can be used to obtain material-specific moisture calibrations, thereby providing flexibility in the design of localized FDR monitoring systems.

The fitted curves represent empirical calibration relationships within the investigated moisture and frequency ranges. Their application to different specimens, surface conditions, or environmental scenarios would require dedicated recalibration or independent validation.

### 4.2. Validation of the Portable TDR Prototype

#### 4.2.1. Time- and Frequency-Domain Comparison in Air

Before testing the moisture-conditioned Carparo specimen, microstrip probe M1 was characterized in air using both the PicoVNA 108 and the developed portable TDR prototype. This preliminary assessment provided a simplified and reproducible condition for comparing the two measurement chains before introducing the additional effects associated with the masonry material, moisture distribution, and probe–specimen coupling.

The comparison was first performed in the time domain. The PicoVNA 108 was operated in TDR mode, while the portable prototype directly acquired the corresponding reflectogram. Both responses were represented as functions of the apparent distance defined in Section 2.2 and superimposed on the same axes. The reflectogram acquired using the prototype was then processed through the time-to-frequency-domain reconstruction procedure described in Section 2.3, and the resulting frequency response was compared with that measured directly using the PicoVNA.

**Figure 13:**
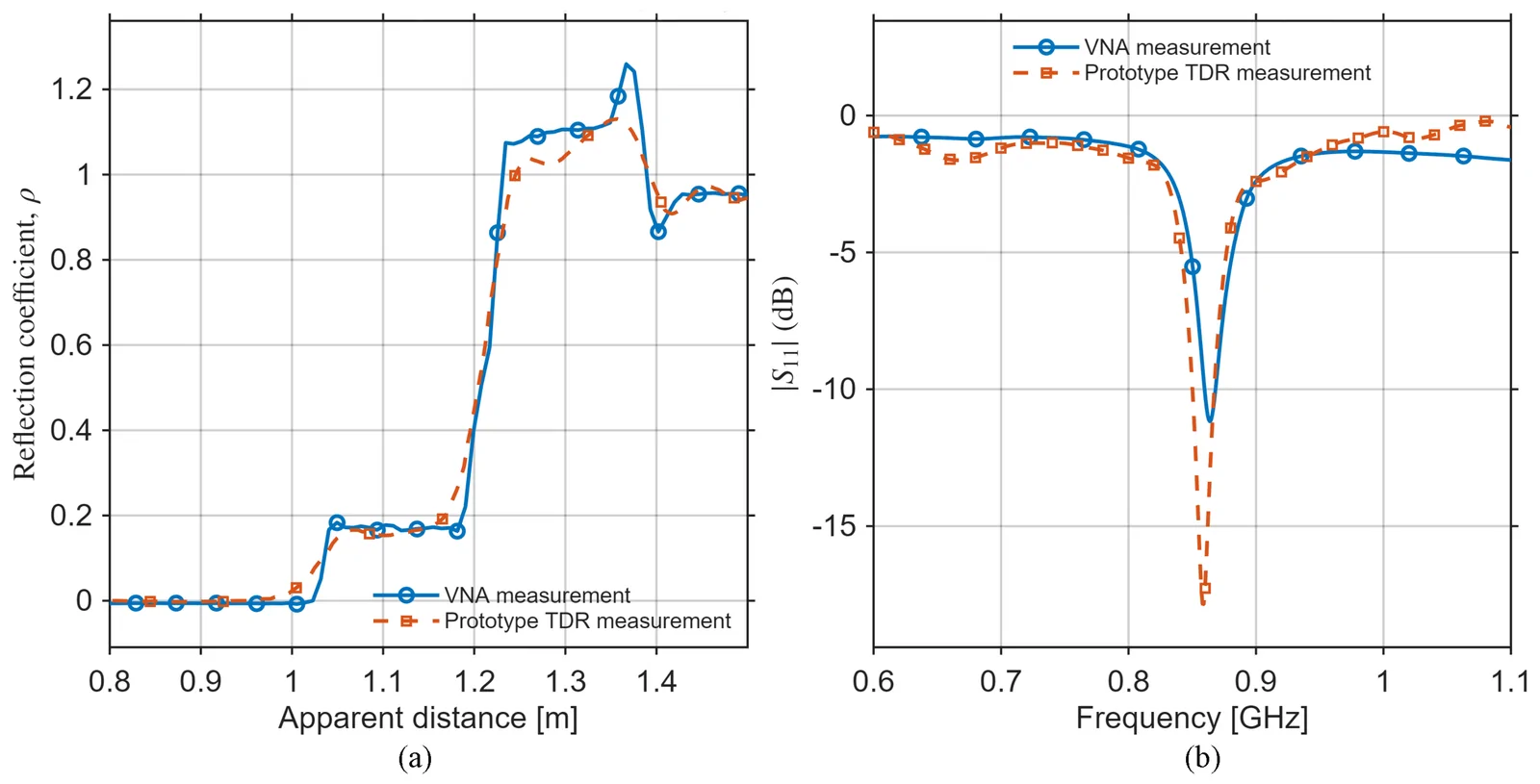
Free-space comparison of microstrip probe M1 using the PicoVNA 108 and the portable TDR prototype: (**a**) superimposed reflectograms acquired using the PicoVNA in TDR mode and the portable prototype, represented as functions of apparent distance; and (**b**) superimposed frequency-domain responses measured directly using the PicoVNA and reconstructed from the prototype-TDR reflectogram.

As shown in Figure 13a, the two reflectograms exhibit a closely corresponding sequence of reflection features along the apparent-distance axis. The principal transitions occur at comparable apparent positions, and the overall waveform evolution is preserved by the portable prototype. Differences are mainly observed in the amplitude of some local maxima and minima, which can be attributed to the different instrumental responses, acquisition chains, and processing procedures. The frequency-domain responses in Figure 13b also show consistent overall behavior. Both measurement chains identify the principal resonance of M1 within the same frequency region, confirming that the time-to-frequency-domain reconstruction preserves the main spectral feature of the probe.

#### 4.2.2. Time-Domain Comparison at Different Moisture Levels

**Figure 14:**
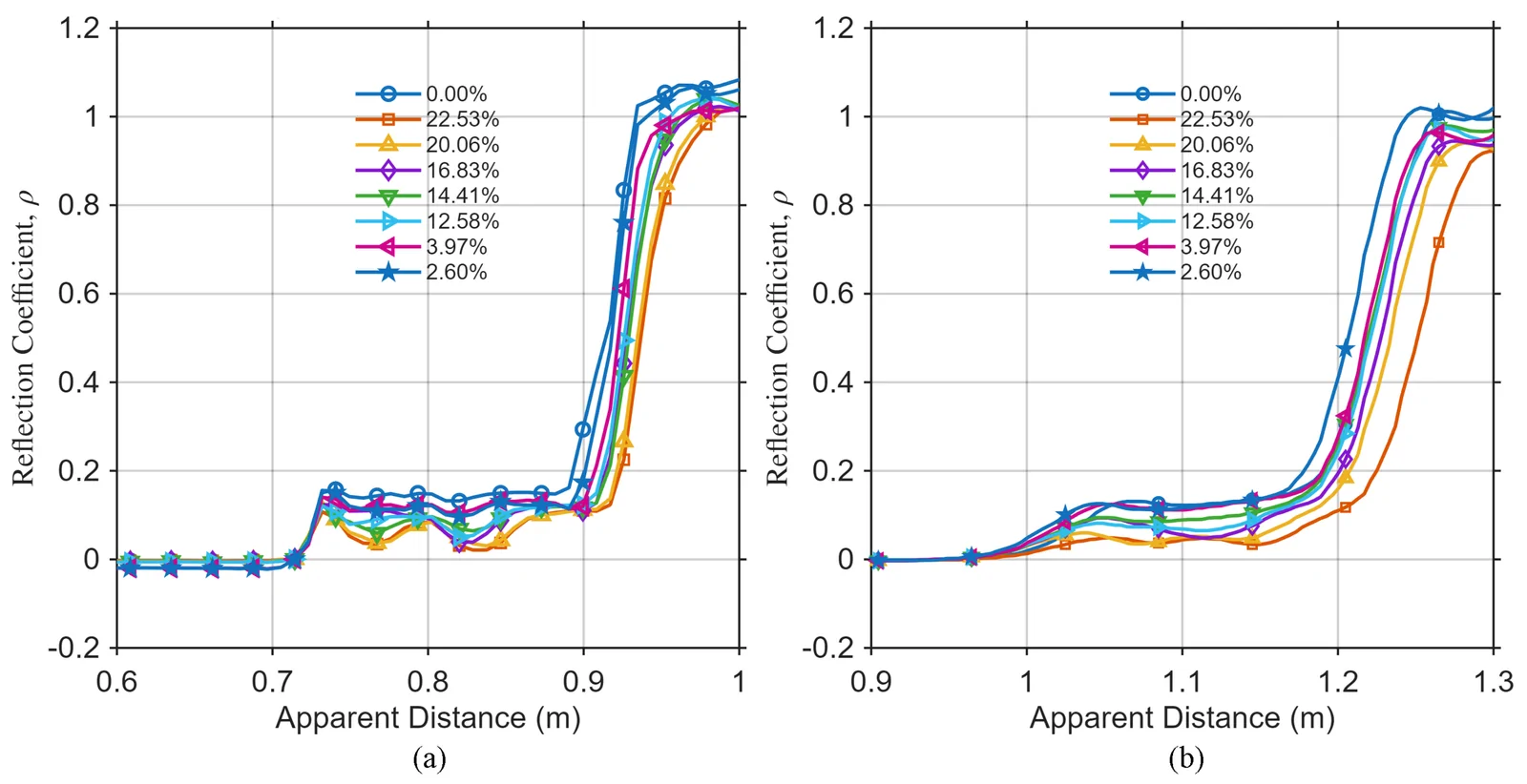
Moisture-dependent time-domain responses of microstrip probe M1 on Carparo: (**a**) reflectograms obtained using the PicoVNA 108 in TDR mode and (**b**) reflectograms acquired using the portable TDR prototype. The same colors and markers identify the corresponding gravimetric moisture levels in both panels.

Figure 14 compares the moisture-dependent time-domain responses obtained using the PicoVNA 108 in TDR mode with the reflectograms directly acquired using the portable TDR prototype on the Carparo specimen. The same colors and markers identify the corresponding gravimetric moisture levels in the two panels, allowing the evolution of the responses during controlled drying to be compared directly.

Both measurement systems reproduced a clear and systematic moisture-dependent evolution of the reflectograms. As the gravimetric moisture content decreased, the principal rising transition shifted toward shorter apparent distances in both panels. The responses therefore preserved the same ordering across the investigated moisture levels, with the dry and low-moisture conditions occurring at the shortest apparent distances and the highest-moisture condition occurring at the longest apparent distance.

The two instruments also exhibited a comparable overall waveform evolution, including an initial low-amplitude region, an intermediate response associated with the probe and connection structure, and the subsequent main transition. These common features confirm that the portable prototype captured the same moisture-sensitive propagation behavior observed using the PicoVNA reference measurement. The difference in the absolute apparent-distance ranges of the two panels is mainly due to the different connection cables used with the two measurement systems. M1 was connected to the PicoVNA 108 through a 30-cm N-to-SMA coaxial cable and to the portable TDR prototype through a 50-cm BNC-to-SMA coaxial cable. The resulting difference in electrical path length introduces a systematic offset along the apparent-distance axis, while the moisture-dependent displacement remains comparable in the two measurements. Nevertheless, the consistent direction and ordering of the moisture-dependent displacement demonstrate that the portable TDR prototype preserves the principal time-domain information associated with moisture variation in the Carparo specimen.

#### 4.2.3. Frequency-Domain Reconstruction at Different Moisture Levels

**Figure 15:**
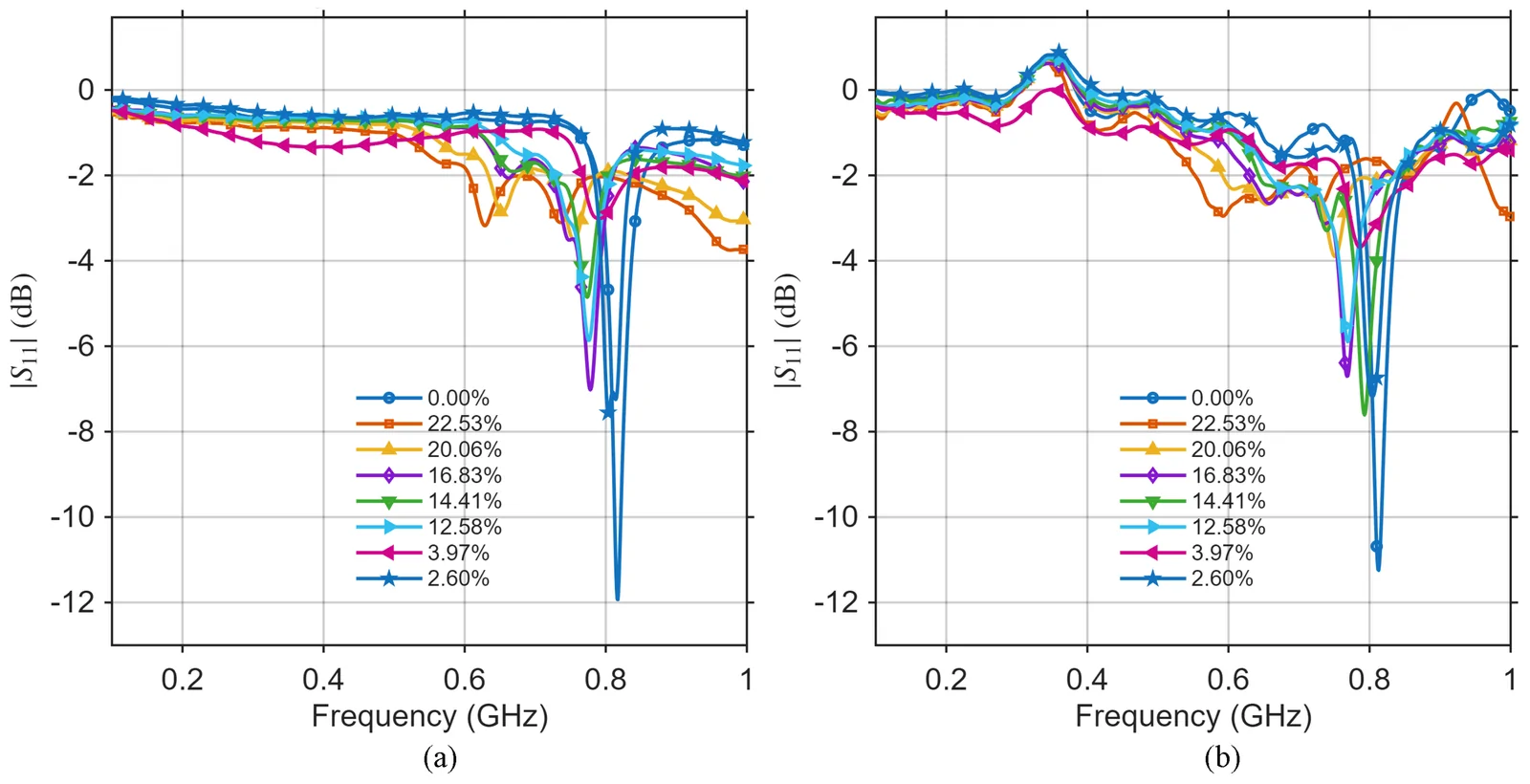
Comparison of the moisture-dependent frequency-domain responses of microstrip probe M1 on Carparo: (**a**) responses measured directly using the PicoVNA 108 and (**b**) responses reconstructed from the reflectograms acquired using the portable TDR prototype. The same colors and markers identify the corresponding gravimetric moisture levels in both panels.

The reflectograms acquired using the portable TDR prototype were processed and calibrated according to the procedure described in Section 2.3. The resulting frequency-domain responses were then compared with those measured directly using the PicoVNA 108 at the corresponding gravimetric moisture levels, as shown in Figure 15.

The resonance frequencies measured directly with the PicoVNA exhibited a clear monotonic shift toward higher frequencies as the gravimetric moisture content decreased. The TDR-reconstructed resonance frequencies generally reproduced the same moisture-dependent evolution, although local deviations from strict monotonicity occurred at the intermediate moisture levels of 16.83%, 14.41%, and 12.58%. As will be shown in the subsequent calibration analysis, these deviations did not prevent the reconstruction from preserving the overall calibration trend. Since the TDR resonance is extracted from a frequency-domain response reconstructed from the measured reflectogram, small variations introduced by the reconstruction, calibration, finite spectral resolution, and resonance-minimum extraction may locally affect its estimated position.

The reconstructed responses also retained the dominant resonance region observed using the PicoVNA over the investigated bandwidth. Although differences remained in the spectral background, resonance depth, and local curve shape, the portable TDR measurement chain consistently recovered the principal moisture-sensitive frequency trend.

To quantify this agreement, the resonance frequency was extracted independently from the PicoVNA-measured and TDR-reconstructed response at each moisture level. The point-by-point differences and the corresponding summary metrics are reported in Table 5.

Across the eight investigated moisture levels, the mean resonance-frequency bias was −2.136 MHz, indicating that the TDR-reconstructed resonance frequencies were, on average, only slightly lower than those measured using the PicoVNA. The standard deviation of the differences was 3.090 MHz, while the MAE and RMSE were 3.315 MHz and 3.594 MHz, respectively. Considering the investigated resonance-frequency range of approximately 733–817 MHz, these values indicate a good overall agreement between the two measurement chains. The limited difference between the RMSE and MAE suggests that the pointwise deviations were relatively uniform, without the presence of pronounced outliers. Overall, the results confirm that the portable TDR prototype successfully preserved the moisture-dependent resonance behavior measured by the PicoVNA, supporting its suitability as a compact and lower-cost solution for repeated masonry-moisture monitoring.

#### 4.2.4. Comparison of Moisture Calibration Curves

**Figure 16:**
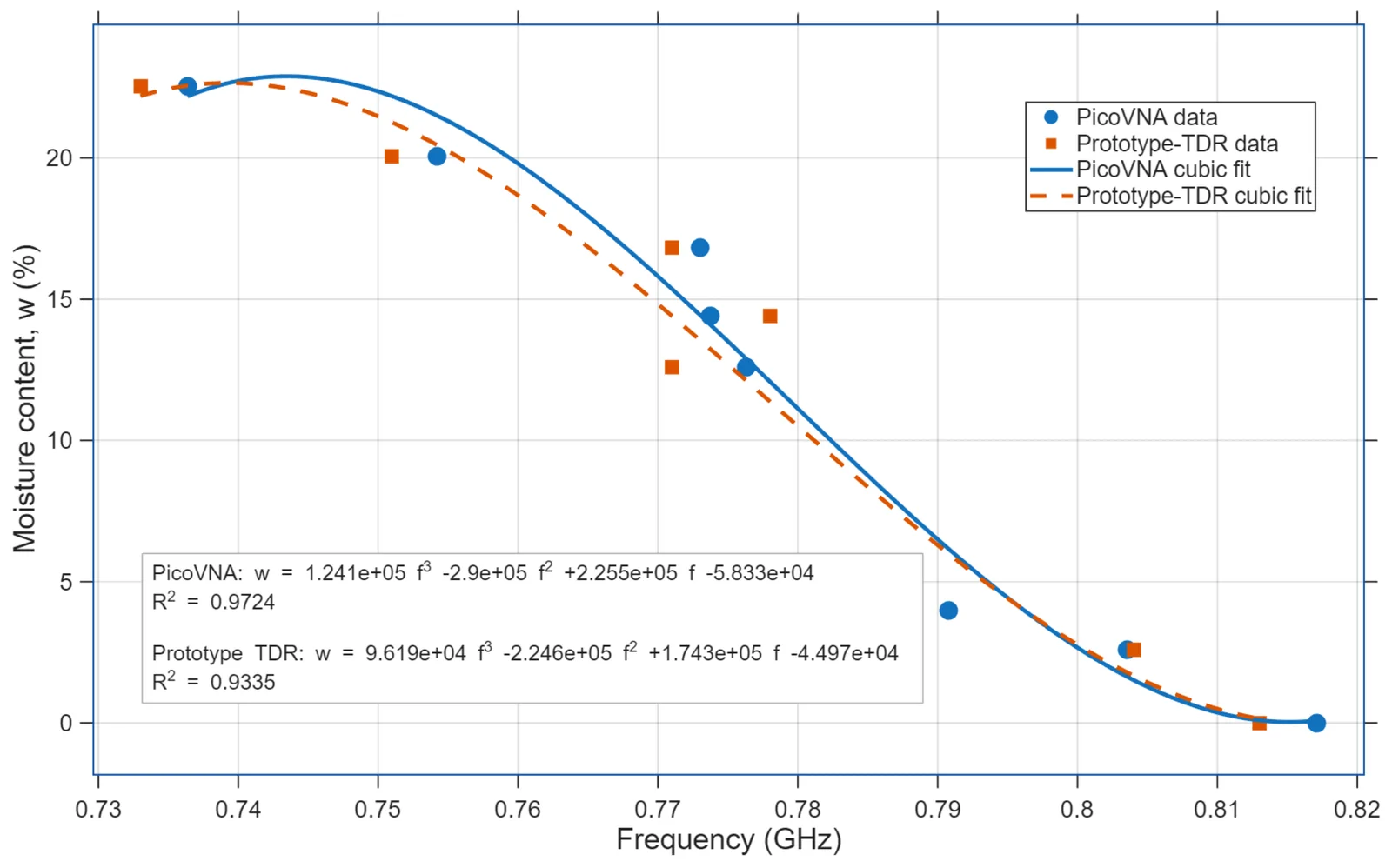
Comparison of the gravimetric moisture–resonance frequency calibration curves obtained for microstrip probe M1 on Carparo. Experimental resonance frequencies measured directly using the PicoVNA 108 and those reconstructed from the portable-TDR reflectograms are shown together with the corresponding third-order polynomial fits. The fitted equations and coefficients of determination, $R^{2}$, are reported in the figure.

As a final step of the validation, two independent moisture–calibration relationships were derived for microstrip probe M1 applied to the Carparo specimen. The first was obtained from the resonance frequencies measured directly using the PicoVNA 108, whereas the second was derived from the resonance frequencies extracted from the frequency-domain responses reconstructed from the portable-TDR reflectograms. In both cases, each resonance-frequency value was associated with the gravimetric moisture content measured at the corresponding drying step. To ensure a consistent comparison between the two measurement chains, the same third-order polynomial model adopted for the FDR characterization was fitted independently to the PicoVNA-measured and TDR-reconstructed datasets. Figure 16 superimposes the experimental points and the corresponding calibration curves, thereby enabling direct evaluation of the moisture-dependent trends obtained using the reference instrument and the developed prototype.

The two fitted curves remained close over most of the experimental interval, showing that the TDR reconstruction preserved the principal moisture-dependent calibration behavior observed using the PicoVNA. The third-order polynomial models achieved coefficients of determination of $R^{2}=0.9724$ for the PicoVNA dataset and $R^{2}=0.9335$ for the TDR-reconstructed dataset. These values indicate that both calibration models provided a suitable representation of the experimental relationship between gravimetric moisture content and resonance frequency. The polynomial coefficients are summarized in Table 6.

Overall, the agreement between the two fitted trends confirms that the resonance frequencies reconstructed from the portable-TDR measurements can be used to derive a moisture-calibration relationship consistent with that obtained using the PicoVNA. This result extends the validation beyond the comparison of individual frequency responses and demonstrates that the developed prototype preserves the information required for resonance-based moisture assessment.

## 5. Conclusions and Future Work

The results support the proposed integrated reflectometric framework, in which different sensing structures and acquisition systems are selected according to the spatial and operational requirements of masonry-moisture monitoring. In both experimental branches, the principal resonance shifted toward higher frequencies as gravimetric moisture decreased, consistently with the reduction in the effective dielectric permittivity of the sensor–material system during drying.

Within this framework, P1, P2, and M1 should not be regarded as interchangeable probes tested under unrelated conditions, but as complementary sensing configurations intended for different applications. The patch–VNA configuration provides accurate and localized moisture characterization based on sensor–material-specific calibration and is particularly suitable for valuable or architecturally significant surfaces, where controlled spatial localization and detailed spectral information are required. Conversely, the elongated microstrip probe combined with the portable TDR prototype offers greater potential for compact, repeated, distributed, and lower-cost monitoring of extended masonry elements, including walls affected by rising damp. The developed prototype is therefore not intended as a universal replacement for VNA measurements, but as a scalable solution for applications in which portability, installation cost, monitoring frequency, and spatial coverage are decisive factors.

The measurements performed using patch antennas P1 and P2 demonstrated that the moisture-dependent resonance response can be observed at different operating frequencies and on different masonry configurations. All six material–antenna datasets produced well-defined nonlinear calibration relationships, with $R^{2}$ values greater than 0.95. The results obtained for plaster on Carparo are particularly relevant because they confirm the applicability of the approach to a layered configuration representative of real masonry surfaces. At the same time, the differences among the fitted curves show that calibration remains specific to the sensor, material, and contact condition.

The second experimental branch focused on the validation of the developed portable TDR prototype using M1 on Carparo. Its contribution within the present framework lies in implementing the established TD–FD processing principles in the dedicated prototype and experimentally validating the complete measurement chain for masonry-moisture assessment against the PicoVNA reference system. The prototype reproduced the principal moisture-dependent evolution observed using the PicoVNA in both the time and frequency domains. The mean resonance-frequency bias was −2.136 MHz, with an SD of the differences of 3.090 MHz, an MAE of 3.315 MHz, and an RMSE of 3.594 MHz. Moreover, the PicoVNA-measured and TDR-reconstructed calibration curves showed comparable nonlinear trends, with $R^{2}=0.9724$ and $R^{2}=0.9335$, respectively.

These results indicate that the prototype preserved not only the principal resonance region, but also the progressive frequency evolution required for moisture calibration.

The present study establishes a solid laboratory validation of the proposed integrated framework, demonstrating consistent moisture sensitivity across different sensing configurations and a close correspondence between the PicoVNA measurements and the responses reconstructed from the portable TDR prototype. The obtained results provide a reliable basis for extending the approach to a broader range of materials, specimens, and operating conditions, including repeated wetting–drying cycles, different contact configurations, surface roughness levels, environmental conditions, and salt contents. In this context, future investigations will also consider amplitude-related characteristics of the reflectometric response, including resonance depth and changes in the overall $|S_{11}|$ response. Since dissolved salts can modify the electrical conductivity and dielectric losses of porous masonry materials, these features may provide complementary information on salt content when analyzed together with the resonance-frequency shift. Dedicated experiments with controlled salt concentrations will be required to assess this potential and to separate salt-related effects from those primarily associated with moisture content. Future developments will build on this validated framework through the design of probes with different lengths and resonance frequencies, improved and repeatable mounting solutions, multichannel interrogation, and automated or remote acquisition. Future developments may also include flexible or conformal planar sensing elements designed to adapt to irregular or curved masonry surfaces, while preserving a stable and reproducible electromagnetic coupling with the investigated material. In parallel, extending the usable bandwidth of the portable TDR system would enable operation at higher frequencies and facilitate the design of more compact sensing elements. These advances could enable scalable and distributed monitoring of moisture evolution along extended masonry walls, while preserving the possibility of using VNA-based measurements for detailed characterization, calibration, and periodic reference assessment.

## Figures and Tables

**Table 1:** Comparison between selected previous works by the authors and the present study.

Work	Application/Material	Sensing Element(s)	Instrumentation	TD–FD Reconstruction	Main Scope/Distinctive Contribution
Ref. [49]	Liquid quality monitoring	Immersive coaxial probe	Commercial TDR and VNA	Yes	Time-to-frequency-domain dielectric characterization of liquids, including compensation of major processing and experimental error contributions and comparison with VNA measurements.
Ref. [50]	Liquid quality monitoring	Immersive coaxial probe	Commercial TDR100 and VNA	Yes	Enhanced combined TD–FD procedure including signal processing, calibration, system modeling, and comparison with direct VNA measurements.
Ref. [39]	Water-content estimation in Cultural Heritage stones	Open-ended coaxial probe, patch resonator, and open-ended waveguide	Commercial TDR100 and VNA	Yes, for the patch-resonator measurements	Comparative microwave moisture assessment on gentile and Lecce stones using different probes and measurement systems; resonance-based relationships were obtained from VNA and TDR measurements.
Ref. [48]	Diffused monitoring in agriculture, pipelines, and building structures	Elongated distributed sensing elements	Commercial portable TDR instruments	No $S_{11}\left(f\right)$ reconstruction	Development and validation of elongated TDR sensing-element networks for spatially distributed detection of dielectric variations.
**Present work**	**Moisture monitoring in historic masonry, including rising-damp scenarios**	**Two contact-based patch antennas (P1, P2) and elongated planar microstrip probe M1**	**PicoVNA 108 and custom-developed low-cost portable TDR prototype**	**Yes**	**Integrated multi-configuration FDR characterization and portable-TDR validation on masonry; six sensor–material FDR datasets including a layered plaster-on-Carparo configuration; time- and frequency-domain comparison with the PicoVNA; and comparison of moisture–resonance calibration relationships.**

**Table 2:** Nominal geometrical and electromagnetic parameters of the patch antennas.

Patch	Nominal Resonance Frequency in Air	Patch Dimensions (W×L)	Substrate Thickness *h*
P1	2.4GHz	40×29.1mm	1.6mm
P2	1.57GHz	45×45mm	1.6mm

**Table 3:** Nominal geometrical and electromagnetic parameters of microstrip probe M1.

Probe	Nominal Resonance Frequency in Air	Conductor Dimensions (W×L)	Substrate Thickness *h*
M1	852.22MHz	2×101.5mm	1.6mm

**Table 4:** Third-order polynomial calibration parameters for the six patch–material combinations, with $f_{\mathrm{res}}$ expressed in GHz.

Material	Patch	*n*	$a_{3}$	$a_{2}$	$a_{1}$	$a_{0}$	$R^{2}$
Lecce stone	P1	10	$-1.22\times 10^{3}$	$7.94\times 10^{3}$	$-1.72\times 10^{4}$	$1.25\times 10^{4}$	0.9585
Lecce stone	P2	10	$-1.55\times 10^{3}$	$6.06\times 10^{3}$	$-7.93\times 10^{3}$	$3.49\times 10^{3}$	0.9902
Carparo	P1	8	$4.71\times 10^{3}$	$-3.25\times 10^{4}$	$7.48\times 10^{4}$	$-5.71\times 10^{4}$	0.9618
Carparo	P2	8	$5.27\times 10^{4}$	$-2.32\times 10^{5}$	$3.42\times 10^{5}$	$-1.67\times 10^{5}$	0.9506
Plaster on Carparo	P1	7	$1.52\times 10^{2}$	$-1.05\times 10^{3}$	$2.36\times 10^{3}$	$-1.73\times 10^{3}$	0.9903
Plaster on Carparo	P2	7	$-1.91\times 10^{3}$	$7.87\times 10^{3}$	$-1.09\times 10^{4}$	$5.08\times 10^{3}$	0.9927

**Table 5:** Comparison between the resonance frequencies measured using the PicoVNA 108 and reconstructed from the portable-TDR responses at the investigated gravimetric moisture levels. The frequency difference was calculated as $\Delta f_{\mathrm{res}}=f_{\mathrm{res}}^{\mathrm{TDR}}-f_{\mathrm{res}}^{\mathrm{VNA}}$.

*w* (%)	$f_{\mathrm{res}}^{\mathrm{VNA}}$ (MHz)	$f_{\mathrm{res}}^{\mathrm{TDR}}$ (MHz)	$\Delta f_{\mathrm{res}}$ (MHz)
22.53	736.374	733.005	−3.369
20.06	754.223	751.005	−3.219
16.83	773.023	771.005	−2.018
14.41	773.773	778.005	4.232
12.58	776.323	771.005	−5.318
3.97	790.772	787.005	−3.767
2.60	803.522	804.005	0.483
0.00	817.121	813.005	−4.116
**Mean bias**	**SD of differences**	**MAE**	**RMSE**
−2.136 MHz	3.090 MHz	3.315 MHz	3.594 MHz

**Table 6:** Third-order polynomial calibration parameters obtained for microstrip probe M1 on Carparo using the PicoVNA-measured and TDR-reconstructed resonance frequencies, with $f_{\mathrm{res}}$ expressed in GHz.

Measurement Chain	*n*	$a_{3}$	$a_{2}$	$a_{1}$	$a_{0}$	$R^{2}$
PicoVNA	8	$1.241\times 10^{5}$	$-2.900\times 10^{5}$	$2.255\times 10^{5}$	$-5.833\times 10^{4}$	0.9724
Prototype TDR	8	$9.619\times 10^{4}$	$-2.246\times 10^{5}$	$1.743\times 10^{5}$	$-4.497\times 10^{4}$	0.9335

## Data Availability

The data presented in this study are available from the corresponding authors upon reasonable request.

## References

[1] Mahato C., Pulatsu B., Spadea S., Lourenço P.B., Cui G., Bompa D., Sagaseta J., Funari M.F. (2026). Impact of Moisture-Related Phenomena on the Mechanical Performance and Deterioration of Historic Masonry: A Systematic Literature Review. Int. J. Archit. Herit..

[2] Ma Y., Xie H., Li Y., Hokoi S., Zhang X., Wang X. (2024). Water-related deterioration risk assessment for sustainable conservation of heritage buildings in the Forbidden City, China. Dev. Built Environ..

[3] Nik V.M., Mundt-Petersen S.O., Kalagasidis A.S., De Wilde P. (2015). Future Moisture Loads for Building Facades in Sweden: Climate Change and Wind-Driven Rain. Build. Environ..

[4] Franzoni E., Berk B., Bassi M., Marrone C. (2023). An integrated approach to the monitoring of rising damp in historic brick masonry. Constr. Build. Mater..

[5] Guolo E., Falchi L., Cimino D., Balliana E., Peron F., Zendri E. (2025). Investigation of capillary rise in brick masonry models under controlled conditions. Constr. Build. Mater..

[6] de Melo M.E.S., de Arimátea Rocha E., de Lima V.M.E., Silva F.A.N. (2026). Diagnostic and characterization techniques for historic masonries with rising damp: A systematic literature review. J. Build. Pathol. Rehabil..

[7] Afif-Khouri E., Lozano-Martínez A., López de Rego J.I., López-Gallego B., Forján-Castro R. (2025). Capillary Rise and Salt Weathering in Spain: Impacts on the Degradation of Calcareous Materials in Historic Monuments. Buildings.

[8] Delgado J.M.P.Q., Guimarães A.S., de Freitas V.P., Antepara I., Kočí V., Černý R. (2016). Salt Damage and Rising Damp Treatment in Building Structures. Adv. Mater. Sci. Eng..

[9] Balksten K., Strandberg-de Bruijn P. (2021). Understanding Deterioration due to Salt and Ice Crystallization in Scandinavian Massive Brick Masonry. Heritage.

[10] Weiss T., Válek J., Slížková Z., Slavík M., Náhunková P., Mareš J., Kozlovcev P. (2025). Migration of Water and Salt in Confined Archaeological Complexes: Comprehensive Investigation Below the Third Courtyard of the Prague Castle. Int. J. Archit. Herit..

[11] Hall C., Hoff W.D. (2011). Water Transport in Brick, Stone and Concrete.

[12] Gaylarde C.C. (2020). Influence of Environment on Microbial Colonization of Historic Stone Buildings with Emphasis on Cyanobacteria. Heritage.

[13] Gómez-Patrocinio F.J., Mileto C., García-Soriano L., Vegas López-Manzanares F. (2020). Material Weathering and Structural Damage in Historic Adobe Constructions in Spain: Preliminary Results of a Quantitative Approach. Stud. Conserv..

[14] Bocca P., Valente S., Grazzini A., Alberto A. (2014). Detachment Analysis of Dehumidified Repair Mortars Applied to Historical Masonry Walls. Int. J. Archit. Herit..

[15] Jiménez-Desmond D., Arizzi A., Cardell C. (2023). A Case Study of Renaissance Wall Paintings in Granada (Spain): Historical–Artistic Analysis, Materials Characterization, and State of Conservation. Minerals.

[16] Torraca G. (2009). Lectures on Materials Science for Architectural Conservation.

[17] Karoglou M., Moropoulou A., Mouzakis C., Asimakopoulos S. (2025). Monitoring of a Historic Building’s Masonry Moisture Content. J. Build. Eng..

[18] Rosina E. (2018). When and How Reducing Moisture Content for the Conservation of Historic Building: A Problem-Solving View or Monitoring Approach?. J. Cult. Herit..

[19] Wojciechowska G., Bednarz Ł.J., Dolińska N., Opałka P., Krupa M., Imnadze N. (2024). Intelligent Monitoring System for Integrated Management of Historical Buildings. Buildings.

[20] Rosina E., Esmaeilian Toussi H. (2025). Microclimate Monitoring Using Multivariate Analysis to Identify Surface Moisture in Historic Masonry in Northern Italy. Appl. Sci..

[21] Robens E., Massen C.H., Hardon J.J. (1994). Studies on Historical Gravimetric Hygrometers. Thermochim. Acta.

[22] Pinchin S.E. (2008). Techniques for Monitoring Moisture in Walls. Stud. Conserv..

[23] Avdelidis N.P., Moropoulou A. (2004). Applications of infrared thermography for the investigation of historic structures. J. Cult. Herit..

[24] Samouëlian A., Cousin I., Tabbagh A., Bruand A., Richard G. (2005). Electrical resistivity survey in soil science: A review. Soil Tillage Res..

[25] Gummerson R.J., Hall C., Hoff W.D., Hawkes R., Holland G.N., Moore W.S. (1979). Unsaturated water flow within porous materials observed by NMR imaging. Nature.

[26] Saarenketo T., Scullion T. (2000). Road evaluation with ground penetrating radar. J. Appl. Geophys..

[27] Knight R. (2001). Ground penetrating radar for environmental applications. Annu. Rev. Earth Planet. Sci..

[28] Gregory A.P., Clarke R.N., Hodgetts T.E., Symm G.T. (2010). RF and microwave dielectric measurements upon materials for moisture sensing applications. Meas. Sci. Technol..

[29] Cataldo A., Farhat I., Farrugia L., Persico R., Schiavoni R. (2022). A Method for Extracting Debye Parameters as a Tool for Monitoring Watered and Contaminated Soils. Sensors.

[30] Suchorab Z., Tabiś K., Brzyski P., Szczepaniak Z., Rogala T., Susek W., Łagód G. (2022). Comparison of the moist material relative permittivity readouts using the non-invasive reflectometric sensors and microwave antenna. Sensors.

[31] Panico S., Herrera-Avellanosa D., Troi A. (2023). Monitoring rising damp in solid masonry walls: An experimental comparison of five different methods. J. Build. Eng..

[32] Kulisz M., Kłosowski G., Rymarczyk T., Hoła A., Niderla K., Sikora J. (2024). The use of the multi-sequential LSTM in electrical tomography for masonry wall moisture detection. Measurement.

[33] Li P., Dong S., Fu F., Xie Q., Jing B., Luo Q., Liu Y., He Y., Zhong N. (2025). Fiber Bragg grating sensor for accurate and in situ monitoring of moisture content of sandstone. Sens. Actuators B Chem..

[34] Kot P., Shaw A., Jones K.O., Cullen J.D., Mason A., Al-Shamma’a A.I. (2014). The Feasibility of Using Electromagnetic Waves in Determining the Moisture Content of Building Fabrics and the Cause of the Water Ingress. Int. J. Smart Sens. Intell. Syst..

[35] Pittella E., Cannazza G., Cataldo A., Cavagnaro M., D’Alvia L., Masciullo A., Schiavoni R., Piuzzi E. (2025). Non-Destructive Permittivity and Moisture Analysis in Wooden Heritage Conservation Using Split Ring Resonators and Coaxial Probe. Sensors.

[36] Robinson D.A., Jones S.B., Wraith J.M., Or D., Friedman S.P. (2003). A review of advances in dielectric and electrical conductivity measurement in soils using TDR. Vadose Zone J..

[37] Topp G.C., Davis J.L., Annan A.P. (1980). Electromagnetic determination of soil water content: Measurements in coaxial transmission lines. Water Resour. Res..

[38] Pittella E., Schiavoni R., Monti G., Masciullo A., Scarpetta M., Cataldo A., Piuzzi E. (2022). Split Ring Resonator Network and Diffused Sensing Element Embedded in a Concrete Beam for Structural Health Monitoring. Sensors.

[39] Piuzzi E., Pittella E., Pisa S., Cataldo A., De Benedetto E., Cannazza G. (2018). Microwave reflectometric methodologies for water content estimation in stone-made cultural heritage materials. Measurement.

[40] D’Alvia L., Pittella E., Rizzuto E., Piuzzi E., Del Prete Z. (2022). A portable low-cost reflectometric setup for moisture measurement in cultural heritage masonry unit. Measurement.

[41] Rizou M.E., Marcelli R., Capoccia G., Proietti E. (2024). Non-destructive microwave techniques for the quantification and elimination of moisture in cultural heritage monuments. J. Cult. Herit..

[42] Dalton F.N., Herkelrath W.N., Rawlins D.S., Rhoades J.D. (1984). Time-domain reflectometry: Simultaneous measurement of soil water content and electrical conductivity. Science.

[43] Heimovaara T.J. (1993). Design of triple-wire time domain reflectometry probes in practice and theory. Soil Sci. Soc. Am. J..

[44] Futa A., Jastrzębska M., Paśnikowska-Łukaszuk M., Wośko E., Suchorab Z. (2023). Improving the Calibration of Surface Time Domain Reflectometry Sensors for Moisture Evaluation of Building Materials Using the Analysis of Covariance Method. Adv. Sci. Technol. Res. J..

[45] Lee D., Kim D.-J., Lee J.-S., Tutumluer E., Byun Y.-H. (2024). Evaluation of electrical resistivity of cement-based materials using time domain reflectometry. Measurement.

[46] Suchorab Z., Widomski M.K., Lagod G., Barnat-Hunek D., Majerek D. (2018). A Noninvasive TDR Sensor to Measure the Moisture Content of Rigid Porous Materials. Sensors.

[47] Suchorab Z., Majerek D., Kočí V., Černý R. (2020). Time domain reflectometry flat sensor for non-invasive monitoring of moisture changes in building materials. Measurement.

[48] Cataldo A., De Benedetto E., Schiavoni R., Tedesco A., Masciullo A., Cannazza G. (2021). Microwave reflectometric systems and monitoring apparatus for diffused-sensing applications. Acta IMEKO.

[49] Cataldo A., Catarinucci L., Tarricone L., Attivissimo F., Trotta A. (2007). A frequency-domain method for extending TDR performance in quality determination of fluids. Meas. Sci. Technol..

[50] Cataldo A., Catarinucci L., Tarricone L., Attivissimo F., Piuzzi E. (2009). A Combined TD–FD Method for Enhanced Reflectometry Measurements in Liquid Quality Monitoring. IEEE Trans. Instrum. Meas..

[51] Nicolson A.M. (1973). Forming the fast Fourier transform of a step response in time-domain metrology. Electron. Lett..

[52] Jha A.K., Akhtar M.J. (2017). Ultra-broadband material spectroscopy from scattering parameters obtained from time-domain measurements. J. Frankl. Inst..

[53] Karoglou M., Mouzakis C., Bakolas A., Asimakopoulos S., Mustapha G. (2024). Monitoring and Calibrating Building Materials Drying Kinetics with Capacitance Sensors. Appl. Sci..

[54] Mikušová D., Suchorab Z., Paśnikowska-Łukaszuk M., Zaburko J., Trnik A. (2024). Applying the Machine Learning Method to Improve Calibration Quality of Time Domain Reflectometry Measuring Technique. Adv. Sci. Technol. Res. J..

[55] Jastrzębska M., Futa A., Mikušová D., Suchorab Z. (2024). The Comparison of One-Variable and Two-Variable Polynomial Regression Models to Measure the Cellular Concrete Moisture Using the Time Domain Reflectometry Method. Adv. Sci. Technol. Res. J..

